# Efficacy of dual KRAS^G12D^–EGFR blockade versus triple combinations in patient-derived models of *KRAS*^G12D^-mutant colorectal cancer

**DOI:** 10.1038/s41419-026-08900-0

**Published:** 2026-05-28

**Authors:** Simonetta M. Leto, Francesco Sassi, Eugenia R. Zanella, Valentina Vurchio, Francesca Cottino, Martina Ferri, Antonella Damico, Elena Grassi, Andrea Bertotti, Livio Trusolino

**Affiliations:** 1 Candiolo Cancer Institute—FPO IRCCS, 10060 Candiolo, Torino, Italy; 2 Department of Oncology, Candiolo Satellite, University of Torino, 10060 Candiolo, Torino, Italy

**Keywords:** Targeted therapies, Cancer models

## Abstract

*KRAS*^G12D^ is one of the most prevalent and treatment-refractory mutations in colorectal cancer (CRC). The emergence of selective KRAS^G12D^ inhibitors offers new therapeutic opportunities, yet preclinical evidence indicates that monotherapy efficacy is constrained by signaling feedback and pathway redundancy, emphasizing the need for rational combination strategies. We systematically evaluated the biochemical, biological, and therapeutic activity of single, dual, and triple regimens combining the KRAS^G12D^ inhibitor MRTX1133 with cetuximab (EGFR inhibitor), alpelisib (PI3Kα inhibitor), or trametinib (MEK inhibitor) in a panel of patient-derived tumoroids and xenografts (PDXs) from metastatic CRC. MRTX1133 displayed mutation-specific activity in *KRAS*^G12D^-mutant tumoroids. Dual blockade of KRAS^G12D^ and EGFR with MRTX1133 + cetuximab achieved near-complete inhibition of MAPK and PI3K-AKT signaling, with only marginal additional suppression upon addition of alpelisib or trametinib. In PDX models, triple combinations conferred no survival advantage over MRTX1133 + cetuximab. Likewise, dual therapy with trametinib + cetuximab was as effective as the triple regimen, suggesting functional redundancy between direct KRAS inhibition and downstream MEK blockade when EGFR is co-targeted. In non-*KRAS*^G12D^-mutant models, MRTX1133 + cetuximab modestly reduced KRAS pathway activity and slightly delayed tumor growth, consistent with a potential ‘leakage effect’. Collectively, our results identify dual KRAS^G12D^ - EGFR inhibition as the regimen delivering maximal pathway suppression and therapeutic benefit in clinically relevant CRC models, with no clear advantage from more complex triple combinations. This work encourages prioritizing KRAS-EGFR co-targeting over multi-agent strategies that risk added toxicity, and provides a strong rationale for advancing KRAS^G12D^ inhibitors + cetuximab as a backbone targeted therapy in future clinical trials for *KRAS*^G12D^-mutant CRC.

## Introduction

RAS GTPases (KRAS, NRAS, and HRAS) are key molecular switches that transduce signals from growth factor receptors to intracellular pathways, promoting cellular growth and proliferation [1, 2]. Mutations in *RAS* genes occur in approximately 20% of human cancers [3] and cluster predominantly at three hotspot positions in exon 2 at codons G12, G13, and Q61 [1, 2].

The development of mutant-selective KRAS inhibitors represents a significant breakthrough in targeted cancer therapy, offering high-affinity inhibition of oncogenic KRAS while sparing the wild-type protein [4]. Based on strong clinical responses observed in single-arm clinical trials, covalent KRAS^G12C^ inhibitors, such as sotorasib and adagrasib, have received FDA accelerated approval for advanced non-small cell lung cancer (NSCLC), where *KRAS*^G12C^ mutations are present in ~12% of cases [4–8]. However, translating this success to colorectal cancer (CRC) has proven challenging. The *KRAS*^G12C^ mutation is present in only ~3% of CRC cases, limiting the field of applicability of mutant-selective KRAS inhibitors in this setting [4]. Additionally, unlike NSCLC, monotherapy with KRAS^G12C^ inhibitors in patients with metastatic CRC (mCRC) has shown limited efficacy [9, 10]. Preclinical studies identified adaptive EGFR upregulation as a compensatory resistance mechanism to KRAS^G12C^ inhibition in CRC [11], prompting clinical investigations into combination strategies with anti-EGFR antibodies (cetuximab or panitumumab). These combinations have demonstrated meaningful clinical activity in the KRYSTAL-1 (NCT03785249) and CodeBreaK 300 (NCT05198934) trials and are now approved for the treatment of chemorefractory mCRC [12–14].

The most common *KRAS* mutation, *KRAS*^*G12D*^, occurs in approximately 40% of pancreatic cancers, 10–12% of CRCs, and 4% of NSCLC cases [15]. Recent preclinical studies have demonstrated the efficacy of novel selective KRAS^G12D^ inhibitors (MRTX1133 and zoldonrasib) in preclinical tumor models of different origin, including pancreas, lung and colon [16–19]. Although both agents exhibited substantial monotherapy activity in pancreatic and lung cancer models, response rates were markedly lower in CRC, with only a minority of models displaying some sensitivity [17, 19]. Notably, MRTX1133-mediated KRAS^G12D^ blockade, when combined with inhibitors of feedback or by-pass pathways such as EGFR and PI3Kα, produced enhanced anti-tumor effects [16, 17], whereas combination strategies with zoldonrasib have not yet been explored [19].

Although these findings suggest promising therapeutic avenues, it is important to acknowledge that combinatorial therapies with MRTX1133 have been primarily tested in CRC cell lines, either in two-dimensional cultures or as xenografts [17, 18]. Conventional cell lines, while widely used, face important limitations due to artificial culture conditions that introduce genetic, epigenetic, and transcriptomic changes, affecting reproducibility and clinical relevance [20]. Moreover, drug screens in CRC cell lines are hindered by the fact that most cultures have been established from primary tumors of treatment-naïve patients, whereas mutant-selective KRAS inhibitors are typically administered to heavily pre-treated patients with metastatic disease.

To address these limitations, we recently established XENTURION (XENografts and TUmoroids for Research In Oncology), an extensive resource of matched mCRC patient-derived xenografts (PDXs) and PDX-derived tumoroids (PDXTs). XENTURION models recapitulate the genetic heterogeneity of mCRC patients, maintain their genomic and transcriptional identity during serial passaging, and display high intra-pair concordance in molecular profiles and therapeutic responses [21]. These attributes make XENTURION a robust platform for preclinical drug evaluation in mCRC.

In this study, we leveraged our in vitro and in vivo patient-derived models to conduct comparative drug efficacy analyses aimed at identifying an optimal combination therapy from several candidates targeting key KRAS-dependent signaling pathways. MRTX1133 was used as a reference compound owing to its earlier availability as an experimental tool inhibitor. Results from this effort highlight the non-inferiority of dual KRAS^G12D^-EGFR blockade compared to other tested combinations, with clinically relevant implications for improving the therapeutic response to KRAS^G12D^ inhibitors in patients with mCRC.

## Results

### Biological response to single-agent KRAS pathway inhibition in *KRAS*^G12D^-mutant PDX-derived tumoroids

Co-targeting KRAS^G12D^ with either EGFR or PI3Kα has been shown to be more effective than *KRAS*^G12D^ inhibition alone in CRC models in vivo [17, 18]. To identify rational combination strategies that could further enhance therapeutic efficacy, we selected cetuximab and alpelisib as inhibitors of EGFR and PI3Kα, respectively, and evaluated three additional targets whose inhibition might potentiate the antitumor activity of MRTX1133. These targets, chosen for their relevance to KRAS signaling and pro-apoptotic function, included the MEK inhibitor trametinib and two pro-apoptotic agents: A-1331852, which targets BCL-XL – whose blockade is synthetic lethal with KRAS pathway inhibition in *KRAS*-mutant cancer cells from different tissue types [22] – and the MCL1 inhibitor S63845, with MCL1 identified as a priority target in *KRAS* mutant CRC [23].

**Fig. 1:**
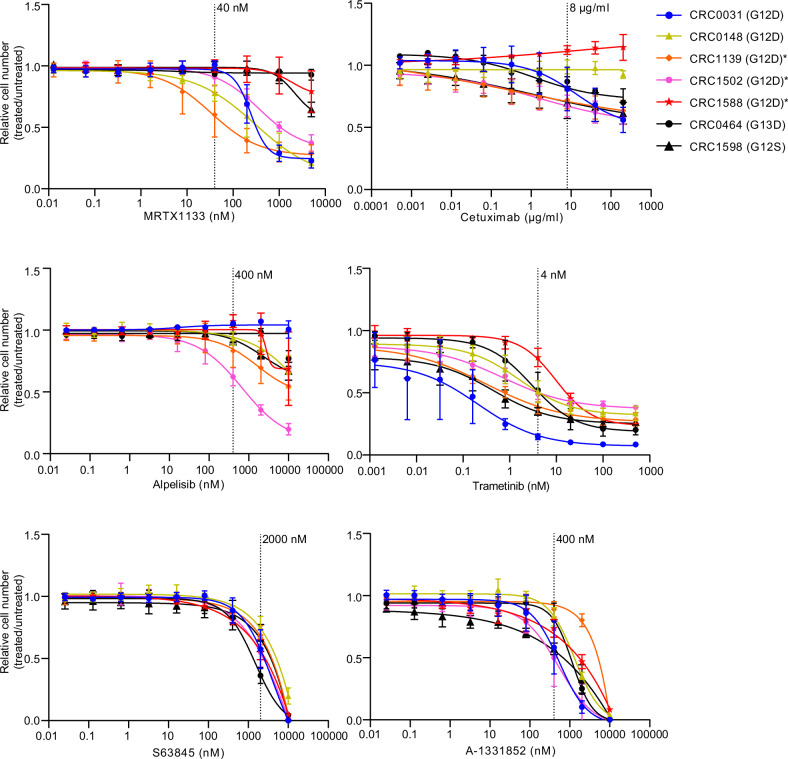
Biological response to single-agent KRAS pathway inhibition in *KRAS*-mutant PDXTs. Quantification of cell viability [measured by adenosine triphosphate (ATP) content] in five *KRAS*^G12D^-mutant and two non-*KRAS*^G12D^-mutant PDXT models treated with the indicated drugs at the specified concentrations for one week. Data are represented as mean values ± SD from three independent experiments, each performed in biological triplicates (*n* = 9). In each graph, dotted lines indicate the tested dose that most closely approximates the lowest IC_50_ value with a good fit calculated across all models. Since *KRAS*-mutant PDXTs showed little or no response to cetuximab, dose-response curves were poorly fitted; therefore, the central dose for subsequent combination screens was selected based on the dose closest to the estimated IC_50_ in the model showing the strongest cetuximab response. Asterisks indicate *PI3KCA*-mutant cases.

To establish optimal drug concentrations for combination screens with MRTX1133, we first assessed the single agent activity of each compound in dose-response assays using five *KRAS*^G12D^-mutant PDXTs (CRC0031, CRC0148, CRC1139, CRC1502, and CRC1588) and two non-*KRAS*^G12D^-mutant PDXTs (CRC0464 and CRC1598, respectively harboring *KRAS*^G13D^ and *KRAS*^*G12S*^ mutations). For subsequent combination studies, we determined a reference central dose for each drug based on the lowest IC_50_ value that exhibited a good fit across all models (Fig. 1).

MRTX1133 was tested at a maximum concentration of 5 μM and titrated down to 0.0128 nM through fivefold serial dilutions. Four of the five *KRAS*^*G12D*^ mutant PDXTs displayed significant impairment of cell viability starting at 40 nM, whereas the non-*KRAS*^G12D^-mutant models were unaffected (Fig. 1 and Supplementary Table 1). Notably, one *KRAS*^G12D^-mutant case (CRC1588) was largely refractory to MRTX1133, displaying a response profile similar to that of the two non-*KRAS*^G12D^-mutant PDXT models. Resistance of *KRAS*^G12D^ tumors to MRTX1133 is expected, as prior studies have reported similar findings in cell lines from CRC and other tumor types [17].

Cetuximab was tested at a maximum concentration of 200 μg/ml, followed by fivefold dilutions to a minimum concentration of 0.512 ng/ml. In agreement with the anticipated resistance of *KRAS* mutant tumors to EGFR inhibition, two PDXTs (CRC0148 and CRC1588) were completely refractory to EGFR inhibition. The remaining five models displayed mild sensitivity at high doses, but none showed a mean reduction in cell viability exceeding 50% (Fig. 1 and Supplementary Table 1). To investigate the molecular basis of this partial cetuximab sensitivity, we analyzed the transcript levels of *EGFR* and its main ligands in all models using available bulk RNAseq data from the XENTURION cohort [21]. In line with pre-clinical and clinical data linking EGFR and ligand expression to cetuximab response in CRC patients [24–28], the two fully resistant models had the lowest expression levels of both *EGFR* and its ligand *EREG* (Supplementary Table 2). Although based on a limited number of models, this observation suggests that *EGFR* and *EREG* expression levels may influence cetuximab sensitivity even in the context of *KRAS* mutant CRC.

The PI3Kα inhibitor alpelisib was tested at a maximum concentration of 10 μM, followed by fivefold dilutions down to 0.0256 nM. It demonstrated moderate efficacy, reducing cell viability in three of the seven models (CRC0148, CRC1139, and CRC1598) at doses starting from 400 nM, and was particularly effective in one additional model (CRC1502) (Fig. 1 and Supplementary Table 1). Notably, the two most sensitive models harbored activating *PIK3CA* mutations (CRC1139: *PI3KCA*^N345K^, CRC1502: *PI3KCA*^E542K^), which are known biomarkers of favorable response to PI3Kα inhibitors, including alpelisib [29, 30]. However, a third model carrying the *PI3KCA*^E545A^ mutation (CRC1588) was resistant to treatment, suggesting that additional factors may modulate sensitivity.

Finally, the MEK inhibitor trametinib was tested across a concentration range spanning 0.5 μM–0.00128 nM using fivefold serial dilutions. As expected, all tested models proved to be dependent on KRAS signaling and displayed marked sensitivity to trametinib, though with some inter-model variability (Fig. 1 and Supplementary Table 1). By contrast, the two pro-apoptotic agents impaired cell viability only at high doses, indicating limited overall activity (Fig. 1 and Supplementary Table 1).

Collectively, these findings indicate that *KRAS* mutant PDXTs exhibited varying degrees of refractoriness to EGFR blockade and heterogeneous susceptibility to PI3K inhibition, while displaying widespread sensitivity to MEK inhibition. Importantly, viability of most *KRAS*^G12D^-mutant PDXTs was markedly reduced by low doses of MRTX1133, confirming the mutation-specific activity of this compound.

### Biochemical response to single-agent KRAS pathway inhibition in *KRAS*^G12D^-mutant PDX-derived tumoroids

Targeting KRAS signaling – either directly with MRTX1133 or indirectly by inhibiting the upstream EGF tyrosine kinase receptor or downstream effectors – impaired cell proliferation in at least one or more *KRAS* mutant models (Fig. 1). To better understand the molecular effects of these treatments, we evaluated drug impact on MAPK/ERK and PI3K/AKT signaling in two *KRAS*^G12D^-mutant PDXTs (CRC0031 and CRC1588) and the two non-*KRAS*^G12D^-mutant models. The *KRAS*^G12D^-mutant models were selected based on their contrasting biological response to MRTX1133, cetuximab, and trametinib (Fig. 1). Both were resistant to alpelisib (Fig. 1), allowing us to assess target inhibition in a context of uniform lack of response.

**Fig. 2:**
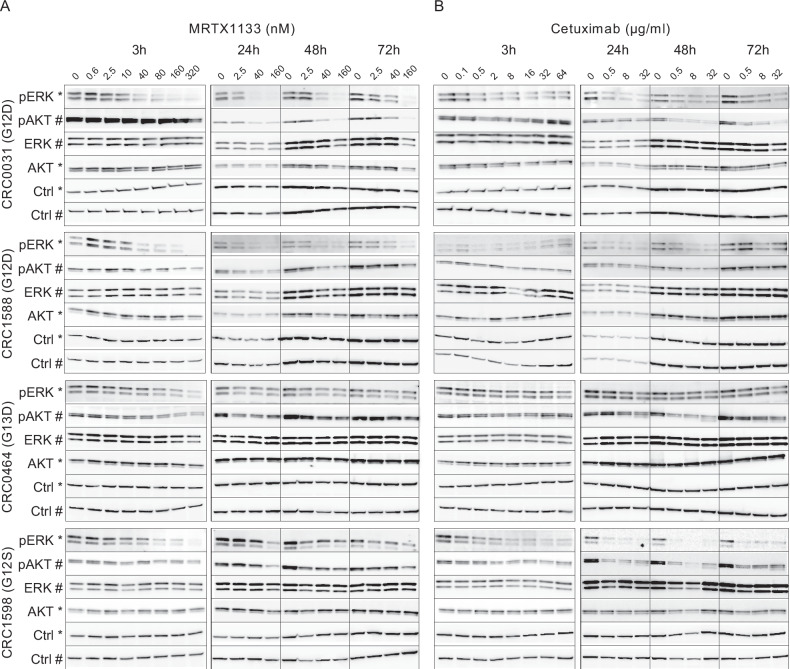
Biochemical response to KRAS^G12D^ or EGFR inhibition in *KRAS*-mutant PDXTs. Western blot analysis of the indicated proteins and phosphoproteins in two *KRAS*^G12D^-mutant and two non-*KRAS*^G12D^-mutant PDXT models. Tumoroids were treated either with a broad concentration range of MRTX1133 (**A**) or cetuximab (**B**) for 3 h, or with a narrower concentration range at multiple time points using the indicated doses. Asterisks and hash symbols denote proteins detected on the same electrophoresis gel. Vinculin was used as the loading control in all experiments except for the time-course assay in CRC0031 PDXT, where α-tubulin was used. Ctrl loading control, pAKT phospho-AKT, pERK phospho-ERK.

**Fig. 3:**
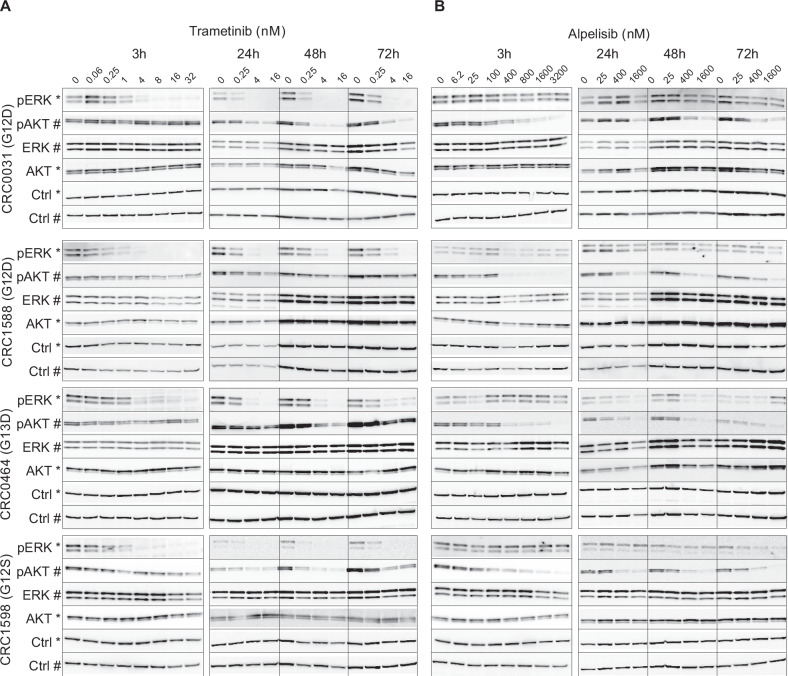
Biochemical response to MEK or PI3Kα inhibition in KRAS-mutant PDXTs. Western blot analysis of the indicated proteins and phosphoproteins in two *KRAS*^G12D^-mutant and two non-*KRAS*^G12D^-mutant PDXT models. Tumoroids were treated either with a broad concentration range of trametinib (**A**) or alpelisib (**B**) for 3 h, or with a narrower concentration range at multiple time points using the indicated doses. Asterisks and hash symbols denote proteins detected from the same electrophoresis gel. Vinculin was used as the loading control in all experiments except for the time-course assay in CRC0031 PDXT, where α-tubulin was used. Ctrl loading control, pAKT phospho-AKT, pERK phospho-ERK.

Tumoroids were treated either with a broad concentration range of MRTX1133, cetuximab, trametinib, or alpelisib for a short exposure (3 h), or with a narrower concentration range at multiple time points (24–72 h); then, ERK and AKT phosphorylation levels were evaluated by western blot (Figs. 2, 3, Supplementary Table 3). In the MRTX1133-resistant *KRAS*^G12D^-mutant model CRC1588, a 3-hour exposure to low-dose MRTX1133 induced a paradoxical increase in ERK phosphorylation, accompanied by concurrent AKT activation (Fig. 2A, Supplementary Table 3). By contrast, the sensitive model CRC0031 exhibited only a slight phospho-ERK rebound without associated AKT hyperactivation (Fig. 2A, Supplementary Table 3). At higher drug concentrations, MRTX1133 led to a dose-dependent decrease of ERK phosphorylation in both models (Fig. 2A, Supplementary Table 3). After 72 hours, relatively high doses of MRTX1133 (40 and 160 nM) reduced phospho-AKT levels more markedly in CRC0031 than in CRC1588 (Fig. 2A, Supplementary Table 3). Thus, MRTX1133 curbed ERK signaling and decreased AKT activity more effectively in CRC0031 than CRC1588, which may explain its differential efficacy in impairing cell viability. In the two non-*KRAS*^G12D^-mutant control models, MRTX1133 also reduced ERK phosphorylation, but required higher doses than in *KRAS*^G12D^-mutant PDXTs, and ERK inhibition over time was less pronounced (Fig. 2A, Supplementary Table 3). Moreover, MRTX1133 exerted negligible effects on AKT phosphorylation, and only at high concentrations. This suggests an off-target effect of MRTX1133 that becomes apparent exclusively at high compound concentrations (Fig. 2A, Supplementary Table 3).

In the *KRAS*^G12D^-mutant model CRC0031, which displayed partial sensitivity to cetuximab, treatment with high antibody doses induced a slight reduction of phospho-ERK levels, whereas no inhibition was observed in the cetuximab-resistant *KRAS*^G12D^-mutant case CRC1588 (Fig. 2B, Supplementary Table 3). Similarly, in time-course experiments, cetuximab exposure resulted in long-lasting suppression of both ERK and AKT phosphorylation in CRC0031 but not in CRC1588 (Fig. 2B, Supplementary Table 3). Among the two non-*KRAS*^G12D^-mutant models, CRC1598 displayed a pronounced, dose-dependent reduction of ERK phosphorylation starting at very low antibody concentration and a durable inhibition of both ERK and AKT over time, while CRC0464 showed a more attenuated response (Fig. 2B, Supplementary Table 3). These findings are consistent with the observed differences in cetuximab sensitivity at the cell viability level (Fig. 1 and Supplementary Table 1).

In the case of trametinib, short-term treatment led to a dose-dependent suppression of phospho-ERK in both *KRAS*^G12D^ models (Fig. 3A, Supplementary Table 3). However, long-lasting inhibition of both ERK and AKT signaling was achieved only in the sensitive case (CRC0031), while the less sensitive model (CRC1588) retained persistent AKT activity (Fig. 3A, Supplementary Table 3). A similar pattern was observed in the non-*KRAS*^G12D^-mutant models, where trametinib effectively reduced ERK phosphorylation but failed to achieve long-lasting AKT suppression (Fig. 3A, Supplementary Table 3), consistent with their intermediate biological sensitivity to the drug. Irrespective of *KRAS* specific mutation and in line with its mechanism of action, alpelisib induced a dose-dependent and long-lasting inhibition of AKT in all models (Fig. 3B, Supplementary Table 3).

In conclusion, these results show an overall correlation between the biological response of each PDXT model to individual drugs and compound ability to inhibit downstream signals. Greater biological sensitivity to treatment was primarily associated with stronger suppressive effects at lower drug doses and/or more durable signal inhibition over time.

### Biological response to dual combination therapies with KRAS pathway inhibitors in *KRAS*^G12D^-mutant PDX-derived tumoroids

**Fig. 4:**
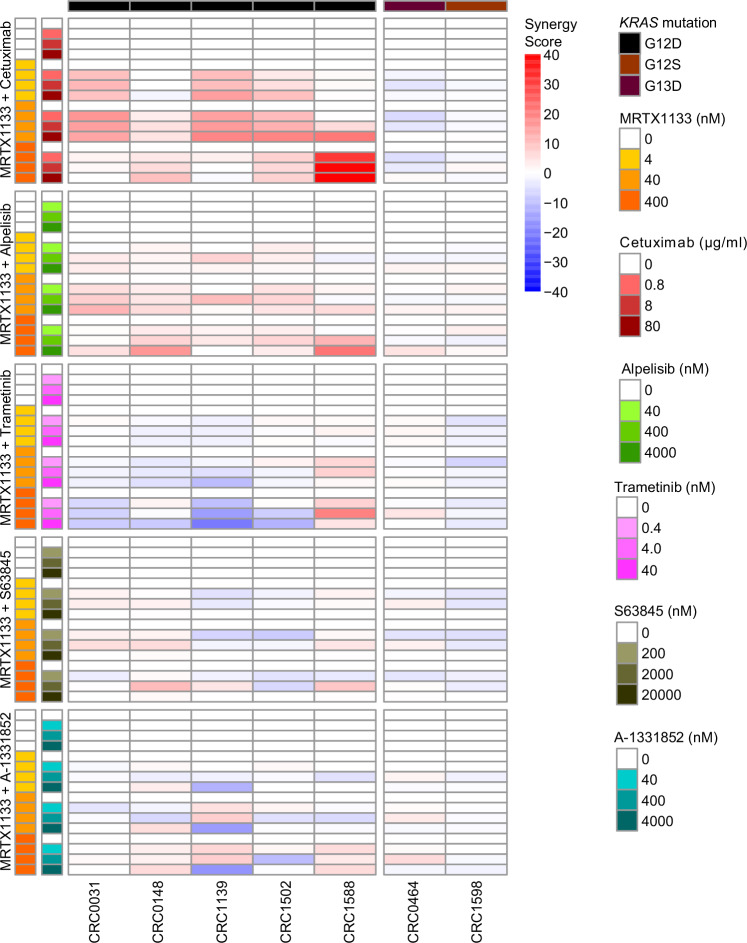
Synergy scores of MRTX1133-based combinations in *KRAS*-mutant PDXTs. Heatmap showing synergy scores for combinations of MRTX1133 with the indicated drugs in seven *KRAS*-mutant PDXT models. Tumoroids were treated for one week with the specified inhibitor concentrations. For each condition, relative cell viability was measured by ATP quantification and normalized to untreated controls. Mean values from three independent experiments, each performed in biological triplicates (*n* = 9), were analyzed using the SynergyFinder R package. Drug combinations with a ZIP synergy score >10 were considered synergistic.

We next explored potential synergistic interactions between MRTX1133 and the other inhibitors in our panel of *KRAS*-mutant PDXT models. Tumoroid lines were either left untreated or exposed to three different doses of each compound for one week, forming a 4 x 4 combination matrix that included a total of nine dual-drug combinations (Fig. 4). As anticipated above, the central dose for each drug was selected based on the lowest IC_50_ value with a good fit from the dose-response curves (Fig. 1). Since all models displayed little or no response to cetuximab, none of the corresponding dose-response curves had a good fit. In this case, the central dose was determined based on the dose closest to the IC_50_ value of the most sensitive model (Fig. 1 and Supplementary Table 1). The highest dose for each drug was 10-fold more concentrated than the central dose, while the lowest dose was 10-fold more diluted (Fig. 4).

Under these conditions, the combination of MRTX1133 and cetuximab demonstrated synergy in four out of five *KRAS*^G12D^-mutant models, consistently across three to six different dual-drug dose combinations (Fig. 4). Similarly, MRTX1133 and alpelisib exhibited synergy in four *KRAS*^G12D^-mutant models, though with lower synergy scores and only at one (in three models) or two (in one model) dual-drug dose combinations (Fig. 4). Trametinib strongly synergized with MRTX1133 in one model and only at a single dose combination (CRC1588) (Fig. 4). In line with their negligible effect as monotherapies at low doses (Fig. 1 lower panels), the MCL1 inhibitor S63845 enhanced MRTX1133 activity in only one model (CRC0148) and with a low synergy score, while the BCL-XL inhibitor A-1331852 failed to synergize with MRTX1133 at any dose combination (Fig. 4).

Of note, none of the tested combinations was synergistic in non-*KRAS*^G12D^-mutant models. Overall, these findings align with previous studies in CRC cell lines showing that both EGFR and PI3Kα inhibitors enhance MRTX1133 ability to impair cell viability in *KRAS*^G12D^-mutant CRC models [17, 18]. Importantly, cetuximab emerged as particularly effective in boosting MRTX1133 efficacy, underscoring its potential as a key component of combinatorial treatment strategies for *KRAS*^G12D^-mutant CRC.

### Biochemical response to PI3Kα or MEK blockade in combination with MRTX1133 and cetuximab in *KRAS*^G12D^-mutant PDX-derived tumoroids

Single-agent biochemical experiments demonstrated that MRTX1133 and cetuximab induced durable inhibition of both ERK and AKT in a biologically sensitive *KRAS*^G12D^-mutant model (Fig. 2, Supplementary Table 3). Additionally, trametinib and alpelisib effectively induced strong and sustained inactivation of their respective targets (Fig. 3, Supplementary Table 3). Based on these findings, we hypothesized that adding either alpelisib or trametinib to the MRTX1133 + cetuximab combination could enhance pathway suppression and achieve broader inhibition of KRAS-dependent signals.

**Fig. 5:**
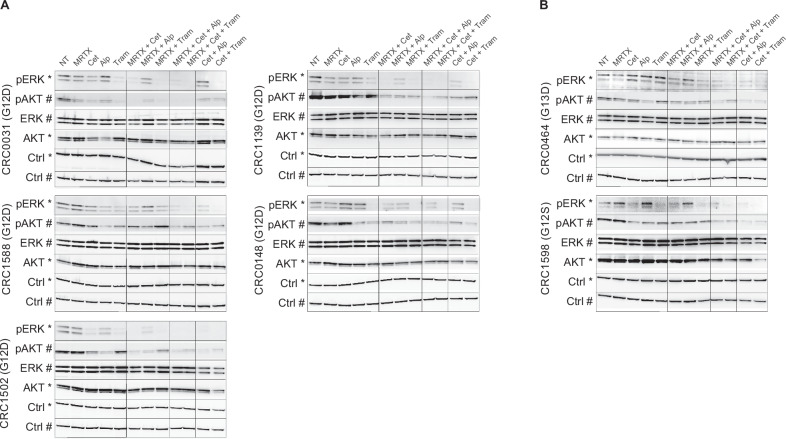
Biochemical response to MRTX1133-based combination therapies in *KRAS*-mutant PDXTs. Western blot analysis of the indicated proteins and phosphoproteins in five *KRAS*^G12D^-mutant (**A**) and two non-*KRAS*^G12D^-mutant (**B**) PDXT models. Tumoroids were treated with the specified modalities for 72 h. Drug concentrations: MRTX1133, 40 nM; cetuximab, 8 μg/ml; alpelisib, 400 nM; trametinib, 4 nM. Asterisks and hash symbols denote proteins detected from the same electrophoresis gel. Vinculin was used as the loading control in all experiments except for the CRC0464 samples marked with an asterisk, where α-tubulin was used. Alp alpelisib, Cet cetuximab, Ctrl loading control, MRTX MRTX1133, NT untreated control, pAKT phospho-AKT, pERK phospho-ERK, Tram trametinib.

To test this hypothesis, we treated the five *KRAS*^G12D^-mutant and the two non-*KRAS*^G12D^-mutant models for 72 h with the following regimens: (i) single-agent MRTX1133, cetuximab, alpelisib, or trametinib; (ii) dual therapy using MRTX1133 as the backbone singlet, combined with either cetuximab, alpelisib, or trametinib; (iii) triple therapy with MRTX1133 + cetuximab as the backbone doublet, combined with either alpelisib or trametinib; (iv) to delineate the distinct contributions of MRTX1133 versus cetuximab in the triple combination, we also tested cetuximab as a backbone singlet, combined with either alpelisib or trametinib.

Western blot analysis revealed residual AKT and/or ERK activity in all models following monotherapy, with some variability across models (Fig. 5A, Supplementary Table 3). Among the MRTX1133-based dual therapies, the combination with cetuximab was generally more effective at reducing both ERK and AKT phosphorylation than MRTX1133 + alpelisib or MRTX1133 + trametinib in four out of five *KRAS*^G12D^-mutant models (Fig. 5A, Supplementary Table 3). Addition of alpelisib or trametinib to the MRTX1133 + cetuximab combination produced only modest further attenuation of signaling, and neither triple therapy achieved complete pathway suppression. (Fig. 5A, Supplementary Table 3). Notably, dual therapy with cetuximab + alpelisib or cetuximab + trametinib was as effective as MRTX1133 + alpelisib or MRTX1133 + trametinib (Fig. 5A, Supplementary Table 3), further underscoring the importance of EGFR co-targeting when intercepting KRAS-dependent signals.

In the two non-*KRAS*^G12D^-mutant control models, MRTX1133-based dual therapy with cetuximab, alpelisib, or trametinib led to a modest but measurable reduction in ERK and AKT phosphorylation. Triple therapy further suppressed both signals. Interestingly, an effect comparable to that exerted by the triple therapy was also observed after exposure to cetuximab in combination with either alpelisib or trametinib, in the absence of MRTX1133 (Fig. 5B, Supplementary Table 3). This suggests that EGFR inhibition by cetuximab “primes” non-*KRAS*^G12D^-mutant tumors for more effective ERK and AKT targeting.

Collectively, these findings highlight the central role of EGFR as a critical co-extinction target in both *KRAS*^G12D^-mutant and non-*KRAS*^G12D^-mutant CRC tumors. Additional blockade of the MAPK or PI3K-AKT pathways provided some incremental suppression of KRAS signaling, but was insufficient to achieve complete pathway inactivation.

### Transcriptional response to PI3Kα or MEK blockade in combination with MRTX1133 and cetuximab in *KRAS*^G12D^-mutant PDX-derived tumoroids

**Fig. 6:**
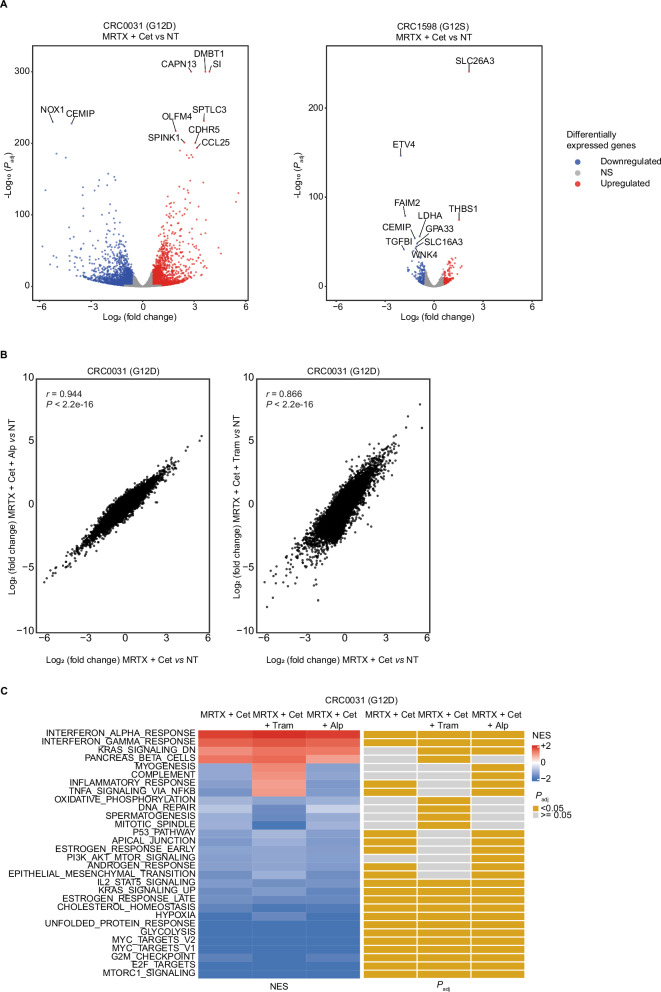
Transcriptional response to MRTX1133-based combination therapies in *KRAS*-mutant PDXTs. **A** Volcano plots showing genes differentially expressed between treated and untreated tumoroids in a *KRAS*^G12D^-mutant model (left) and a non-*KRAS*^G12D^ control model (right) following treatment with MRTX1133 + cetuximab. Genes with an absolute fold change ≥1.5 and an adjusted *P* value <0.05 (Wald test implemented in DESeq2) are highlighted in color, whereas gray dots indicate genes without significant differential expression. **B** Correlation analysis comparing gene expression changes induced by MRTX1133 + cetuximab with those observed after triple therapy including alpelisib (left) or trametinib (right) in *KRAS*^G12D^-mutant tumoroids. Statistical analysis by Pearson’s correlation test. **C** GSEA heatmap showing Hallmark gene sets differentially enriched in *KRAS*^G12D^-mutant tumoroids treated with the indicated therapies relative to untreated controls. Positive and negative normalized enrichment scores indicate gene sets that are upregulated or downregulated, respectively, in treated tumoroids compared with untreated samples. For each gene set and treatment condition, statistical significance (adjusted *P* < 0.05) is indicated in the right panel. Alp alpelisib, Cet cetuximab, MRTX MRTX1133, NES normalized enrichment score, NS nonsignificant, NT untreated control, *P*_adj_ *P* adjusted, Tram trametinib.

To evaluate how dual and triple therapeutic strategies reshape the tumor transcriptome and to identify gene programs associated with response, we performed RNA sequencing (RNA-seq) in one *KRAS*^G12D^-mutant PDXT model (CRC0031) and one non-*KRAS*^G12D^ model (CRC1598). Differential gene expression analysis comparing CRC0031 tumoroids treated with MRTX1133 + cetuximab to untreated controls revealed extensive transcriptional remodeling, with 1958 genes significantly upregulated and 2110 genes downregulated upon treatment (Fig. 6A and Supplementary Table 4). Conversely, in accordance with the minimal biological activity of MRTX1133 + cetuximab in non-*KRAS*^G12D^ models, treatment of CRC1598 resulted in modulation of only 366 genes (175 upregulated and 191 downregulated) (Fig. 6A and Supplementary Table 4).

Correlation analysis demonstrated that the transcriptional changes induced by MRTX1133 + cetuximab were highly concordant with those observed following triple therapy with either alpelisib or trametinib, although the addition of trametinib produced a more pronounced transcriptomic perturbation than alpelisib (Fig. 6B and Supplementary Table 4). These findings align with our biochemical analyses showing that triple therapy only marginally enhances suppression of KRAS signaling compared with MRTX1133 + cetuximab and that trametinib is more effective than alpelisib in inhibiting ERK phosphorylation (Fig. 5).

The close similarity between dual and triple therapy was further confirmed by gene set enrichment analysis (GSEA). Most gene signatures significantly enriched after triple therapy were likewise enriched after treatment with MRTX1133 + cetuximab (Fig. 6C and Supplementary Table 4), indicating that the biological consequences of dual and triple therapy largely overlap. Functional interrogation of these transcriptional programs revealed that gene sets significantly downregulated across all treatment conditions were predominantly associated with cell cycle progression, including MYC and E2F targets and G2/M checkpoint pathways, as well as anabolic metabolic processes (Fig. 6C and Supplementary Table 4). These results are consistent with the established role of oncogenic KRAS signaling in sustaining aerobic glycolysis and lipid and cholesterol biosynthesis [31]. As expected, gene signatures indicative of KRAS pathway suppression were significantly enriched in treated cells (Fig. 6C and Supplementary Table 4). Interestingly, both dual and triple therapy also led to significant enrichment of interferon-α and interferon-γ response signatures (Fig. 6C and Supplementary Table 4). This observation parallels findings in *KRAS*^G12C^-mutant lung tumors, where KRAS inhibition promotes interferon signaling through suppression of MYC activity [32].

Taken together, these data indicate that combined KRAS^G12D^ and EGFR blockade induces a broad transcriptional program consistent with effective KRAS pathway suppression, while the addition of downstream pathway inhibitors produces only incremental transcriptional effects, reinforcing the notion that dual inhibition already captures the dominant biological outcomes of KRAS pathway disruption.

### Response to PI3Kα or MEK blockade in combination with MRTX1133 and cetuximab in *KRAS*^G12D^-mutant PDXs

Biochemical analyses showed that triple therapy was marginally more effective than MRTX1133 + cetuximab in dampening KRAS signaling, yet still fell short of achieving full pathway suppression. To assess whether multi-target vertical approaches could enhance therapeutic efficacy in vivo, we treated three *KRAS*^*G12D*^-mutant PDX models (CRC0031, CRC1588 and CRC1139) with dual regimens combining MRTX1133 with cetuximab, alpelisib, or trametinib, as well as with triple regimens incorporating alpelisib or trametinib into the MRTX1133 + cetuximab backbone (Supplementary Table 5). Survival analyses were conducted until tumors reached a volume of 1000 mm^3^, which served as the study endpoint.

**Fig. 7:**
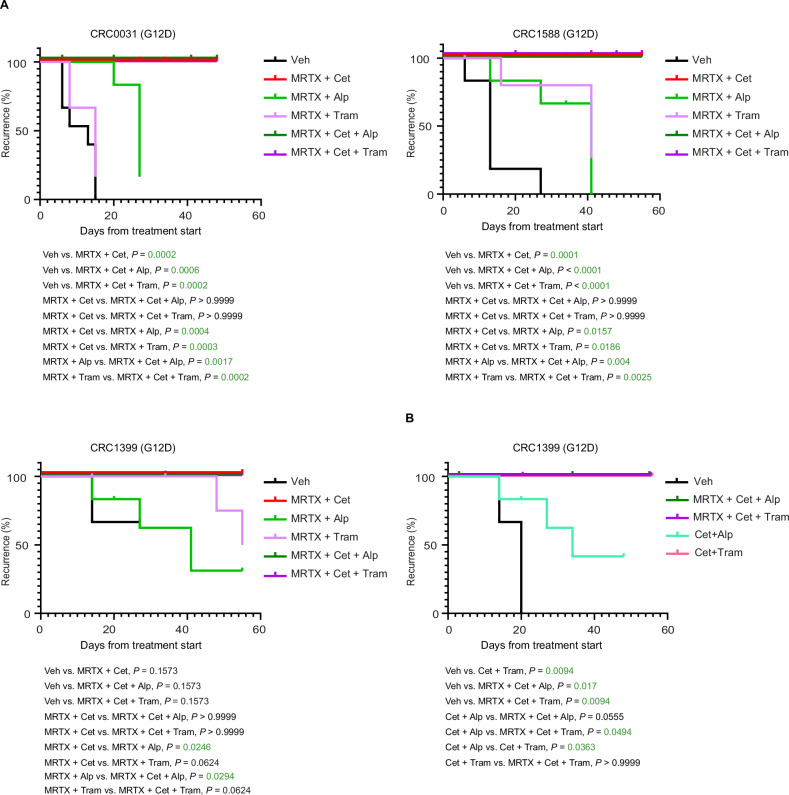
Therapeutic efficacy of MRTX1133-based combination therapies in *KRAS*^G12D^-mutant PDXs. Kaplan-Meier survival curves for mice treated with dual or triple combination therapies incorporating MRTX1133 as the backbone (**A**) or with selected dual and triple regimens, with or without MRTX1133 (**B**). *n* = 3 animals for the vehicle group of CRC1139 and 6–12 animals for all other treatment arms. Drug dosing: cetuximab, 20 mg/kg (intraperitoneal injection, twice weekly); MRTX1133, 10 mg/kg (intraperitoneal injection, twice daily on a 5-days-on/2-days-off schedule); alpelisib, 30 mg/kg (oral gavage, daily), trametinib, 1 mg/kg (oral gavage, daily). Statistical analysis by log-rank (Mantel-Cox) test. Alp alpelisib, Cet cetuximab, MRTX MRTX1133, Tram trametinib, Veh vehicle.

All monotherapies were largely ineffective, producing only minor tumor growth delays in selected models (Supplementary Table 5). Among the dual therapies, MRTX1133 + cetuximab provided the greatest survival benefit, consistently outperforming MRTX1133 + alpelisib and MRTX1133 + trametinib across models, despite some inter-model variability (Fig. 7A and Supplementary Table 5). This aligns with the higher synergy scores and stronger suppression of ERK and AKT phosphorylation demonstrated by MRTX1133 + cetuximab relative to the other dual therapies in tumoroids (Figs. 4 and 5). Notably, the addition of either alpelisib or trametinib to MRTX1133 + cetuximab did not further enhance therapeutic efficacy (Fig. 7A and Supplementary Table 5), consistent with the incomplete suppression of KRAS signaling observed in tumoroids treated with the triple therapy (Fig. 5).

To deconvolute the specific role of MRTX1133 within these combination therapies, we compared the triple therapies with their corresponding dual therapies lacking MRTX1133 in one *KRAS*^G12D^-mutant model (CRC1139). Consistent with the results in Fig. 7A, MRTX1133-containing triple therapies conferred a clear survival benefit (Fig. 7B and Supplementary Table 5). Importantly, the advantage of triple therapy with MRTX1133, cetuximab and trametinib was no greater than that achieved with the dual combination of cetuximab + trametinib (Fig. 7B). We also note that dual therapies with either MRTX1133 + cetuximab or trametinib + cetuximab were similarly effective and sufficient to sustain long-term survival (compare Fig. 7A, B). These findings point to functional redundancy between direct *KRAS*^G12D^ inhibition and downstream MEK inhibition when EGFR is co-targeted. Conversely, downstream PI3Kα inhibition with alpelisib in combination with cetuximab resulted in rapid tumor progression (Fig. 7A and Supplementary Table 5), highlighting the MAPK pathway rather than the PI3K-AKT axis as the primary vulnerability in *KRAS*^G12D^ mutant CRC.

The pharmacodynamic analysis of treated PDXs further supported these findings. MRTX1133 + cetuximab strongly inhibited the phosphorylation of ERK and S6 (a downstream target of AKT), and this suppression was not further enhanced by the addition of alpelisib or trametinib (Supplementary Fig. 1). Likewise, cetuximab + trametinib was as effective as the triple therapy with MRTX1133, cetuximab and trametinib in abating phospho-ERK and phospho-S6 levels in vivo, whereas cetuximab + alpelisib only partly reduced ERK and S6 phosphorylation (Supplementary Fig. 1).

**Fig. 8:**
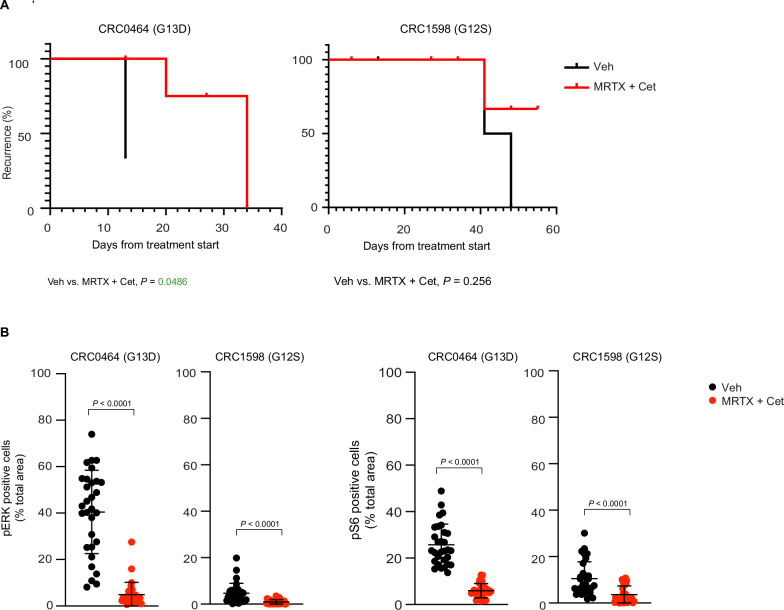
Therapeutic efficacy and pharmacodynamic effect of MRTX1133-based combination therapies in non-*KRAS*^G12D^-mutant PDXs. **A** Kaplan-Meier survival curves for mice treated with the indicated modalities. *n* = 3 animals for the vehicle group, 6 animals for the MRTX1133 + cetuximab group. Drug dosing: cetuximab, 20 mg/kg (intraperitoneal injection, twice weekly); MRTX1133, 10 mg/kg (intraperitoneal injection, twice daily on a 5-days-on/2-days-off schedule). Statistical analysis by log-rank (Mantel-Cox) test. Veh, vehicle; MRTX, MRTX1133; Cet, cetuximab. **B** Morphometric quantification of phospho-ERK and phospho-S6 in the PDX tumors shown in (**A**), treated for 24 h with the specified modalities. At endpoint, three tumors from three different mice per group were explanted and subjected to immunohistochemical analysis. Each dot represents the value measured in one optical field (20x), with 10 fields per tumor depending on the extent of section area (*n* = 30). The plots show mean values ± SD. Statistical analysis by unpaired *t* test with Welch’s correction. Cet cetuximab, MRTX MRTX1133, pERK phospho-ERK, pS6 phospho-S6, Veh vehicle.

In non-*KRAS*^G12D^ mutant PDX models, MRTX1133 + cetuximab induced some delay in tumor growth, especially for CRC0464, which resulted in a minor survival advantage (Fig. 8A and Supplementary Table 5). This partial biological sensitivity is in agreement with the modest reduction in phospho-ERK and phospho-AKT levels observed in corresponding tumoroids treated with MRTX1133 + cetuximab (Fig. 5B) and with the decrease in ERK and S6 phosphorylation detected in treated PDXs (Fig. 8B). These results also point to a potential ‘leakage effect’, whereby *KRAS*^G12D^ mutant-specific inhibition attenuates downstream effector activation even in non-*KRAS*^G12D^-mutant tumors when EGFR is concurrently blocked.

## Discussion

*KRAS* mutations continue to represent a formidable therapeutic hurdle in CRC, and among these, the *KRAS*^G12D^ variant is particularly prevalent and refractory to conventional treatment strategies [33]. Recent advances in the development of mutant-specific KRAS^G12D^ inhibitors have sparked considerable enthusiasm for targeting this mutation [34], but emerging evidence in CRC indicates that monotherapy may not be sufficient to achieve durable responses, owing to complex feedback mechanisms inherent in KRAS signaling networks. This limitation may be especially relevant for MRTX1133, whose reversible, non-covalent binding to the KRAS^G12D^ allosteric pocket [35, 36], combined with suboptimal pharmacokinetic properties, likely contributed to its early withdrawal from clinical deployment. However, zoldonrasib, a covalent and irreversible KRAS^G12D^ inhibitor currently being tested in clinical trials, has likewise shown very limited activity in CRC models compared with other tumor types [19], further underscoring the need for rational combination strategies to extend the therapeutic reach of KRAS^G12D^ blockade to CRC. Chemical genomics-based combinatorial screens and drug-anchored CRISPR-Cas9 synthetic lethality approaches identified EGFR/HER family members and PI3K signaling as collateral dependencies that mitigate response to KRAS^G12D^ inhibition in CRC cell lines. Accordingly, co-targeting EGFR or PI3K enhanced response to *KRAS*^G12D^ blockade in cell culture and xenograft models [17, 18].

While these findings expose the limitations of KRAS^G12D^ inhibitors as single agents and propose combinatorial targets to improve therapeutic outcomes, optimizing drug combination efficacy while balancing potential toxicity remains a critical challenge. The clinical experience with *BRAF*-mutant CRC illustrates this tension well: vertical blockade using triple therapies targeting EGFR, BRAF, and MEK yielded higher response rates and improved overall survival compared with dual blockade of BRAF and EGFR, but at the cost of significant toxicity and reduced quality of life [37, 38]. Weighing therapeutic benefit against cumulative toxicity is therefore essential to inform clinical decision-making.

In this study, we systematically evaluated single, dual, and triple combinations of MRTX1133 with clinically approved inhibitors of EGFR (cetuximab), PI3Kα (alpelisib), and MEK (trametinib) in patient-derived preclinical models, including tumoroids and PDXs. These models more accurately recapitulate the molecular characteristics and heterogeneity of human CRC compared to conventional cell lines [39], underscoring the relevance of our findings for translational applications.

A central result of our work is the demonstration that dual therapy with MRTX1133 and cetuximab is not inferior to triple therapy regimens that incorporate additional PI3Kα or MEK blockade. Biochemically, the combination of MRTX1133 with cetuximab achieves near-maximal inhibition of the MAPK and PI3K-AKT pathways. Beyond this plateau, further addition of alpelisib or trametinib yields only marginal further suppression, suggesting that the incremental benefits of triple combinations may not outweigh the added complexity and potential toxicity. The critical role of EGFR inhibition in sensitizing CRC tumors to KRAS^G12D^ inactivation aligns with the notion that KRAS^G12D^ retains some cycling between the active and inactive states, unlike the largely GDP-locked KRAS^G12C^ mutant, and may therefore be more susceptible to tonic signaling from upstream EGFR activity [40]. This finding has important clinical implications, as it suggests that additional pathway inhibitors may not provide meaningful therapeutic benefits beyond what is achieved with KRAS and EGFR co-targeting.

Phase I studies have shown that patients with *KRAS* mutant CRC treated with EGFR/HER inhibitors and trametinib experience some degree of disease control; however, major toxicity precludes long-term treatment or necessitates intermittent dosing schedules [41, 42]. We found that dual therapy with trametinib + cetuximab was as effective as triple regimens, mirroring the results obtained with MRTX1133 + cetuximab. This suggests that direct KRAS inhibition and downstream MEK blockade exert overlapping therapeutic effects when EGFR is concomitantly targeted. These findings support the use of MRTX1133 for treating *KRAS*^G12D^-mutant CRC tumors, achieving comparable therapeutic benefits as MEK inhibitors while providing greater selectivity and reducing the systemic toxicity associated with MAPK pathway inhibition in normal cells.

Another insight from our study is the ‘leakage effect’ observed in non-*KRAS*^G12D^-mutant CRC models. Although MRTX1133 is designed for selective inhibition of KRAS^G12D^, its combination with cetuximab produced modest tumor growth delays even in tumors lacking this mutation. This was accompanied by a reduction in ERK and AKT phosphorylation, implying that KRAS^G12D^-specific inhibition indirectly or non-selectively dampens downstream signaling in tumors harboring other *KRAS* mutations when EGFR is concurrently inhibited. Notably, experiments in isogenic cell lines have documented modest off-target activity of MRTX1133 against other *KRAS* mutants as well as wild-type *KRAS* protein [43].

Despite the promise of dual combination therapies, a limitation of our study is that the best observed response in vivo was tumor stabilization and prolonged survival rather than robust tumor regression. One potential explanation is that our experiments were conducted in immunocompromised mice, which lack T-cell-mediated immune responses. Growing evidence indicates that the full antitumor efficacy of KRAS inhibitors is closely linked to immune activation. In particular, recent studies have shown that KRAS inhibitors can enhance antigen presentation and promote T-cell infiltration in immunocompetent models, thereby synergizing with immune checkpoint blockade [9, 16, 44–47]. Future investigations incorporating immunocompetent systems or patient-derived co-culture models will be crucial to fully capture the therapeutic potential of KRAS inhibitors in clinically relevant settings.

In summary, our study provides preclinical evidence that dual therapy with MRTX1133 and cetuximab constitutes a rational and clinically viable strategy for *KRAS*^G12D^-mutant CRC. Our findings demonstrate that this combination achieves near-maximal pathway inhibition, comparable to that of more complex triple therapies. Although the clinical development of MRTX1133 was discontinued prematurely, we hypothesize that similar mechanisms may underlie the limited efficacy observed with other KRAS^G12D^ inhibitors, such as zoldonrasib, in CRC preclinical models. These results emphasize the central role of EGFR in modulating resistance mechanisms and support the continued exploration of simplified combination regimens in patient-derived models. Ultimately, our data lay the groundwork for more effective and manageable therapeutic options for patients with *KRAS*-mutant CRC.

## Materials and methods

### Patient-derived tumoroid cultures

Tumoroids of human CRC liver metastases were established from PDX explants. Tumor specimens (0.5 cm × 0.5 cm) were chopped with a scalpel and washed with PBS. Following centrifugation, the final cell preparation was embedded in Growth Factor Reduced Matrigel® (Corning) or Cultrex Basement Membrane Extract (BME Type II or Ultimatrix RGF BME, R&D Systems) and dispensed onto 24-well plates (Corning). After 10–20 min at 37 °C, culture medium was added. Complete tumoroid medium composition was the following: Dulbecco’s modified Eagle medium/F12 supplemented with penicillin-streptomycin, 2 mM L-glutamine, 1mM n-Acetyl Cysteine, B27 (Thermo Fisher Scientific), N2 (Thermo Fisher Scientific) and 20 ng/ml EGF (Sigma-Aldrich). Tumoroids were tested for Mycoplasma and maintained at 37°C in a humidified atmosphere containing 5% CO_2_. Periodic checks of sample identity with the original human specimen (liver metastasis and, when available, normal liver) were performed using a 24 SNP custom genotyping panel (Diatech Pharmacogenetics), and results were analyzed using the MassARRAY Analyzer 4 (SEQUENOM® Inc, California). Culture expansion and biobanking were managed using the Laboratory Assistant Suite [48].

### Viability assays

Pharmacologic experiments were performed in 96-well plates with a thin layer of 50% Matrigel culture medium in the absence of EGF. PDXTs were washed with PBS, incubated with trypsin-EDTA solution for 5 min at 37 °C, and vigorously pipetted to obtain a single cell suspension. Cells were seeded in 2% Matrigel culture medium at a confluence of 5000 cells/well in the absence of EGF. After 2 days from seeding, PDXTs were treated with the modalities indicated in the figure legends. Cetuximab was provided by the healthcare business of Merck KGaA, Darmstadt, Germany; all other small-molecule inhibitors were purchased from MedChemExpress. Cell viability was measured by ATP content using the Cell Titer-Glo luminescent assay kit (Promega).

### Western blot analysis

After 1 day from seeding, PDXTs were treated with the modalities indicated in the figure legends. Proteins were extracted with cold EB buffer (50 mmol/L Hepes pH 7.4, 150 mmol/L NaCl, 1% Triton X-100, 10% glycerol, 5 mmol/L EDTA, 5 mmol/L EGTA) in the presence of phosphatase and protease inhibitors (Thermo Fisher Scientific). Total proteins were quantified using the BCA Protein Assay Reagent Kit (Thermo Fisher Scientific), electrophoresed on precast polyacrylamide gels (Invitrogen) and transferred onto nitrocellulose membranes using a Trans-Blot Turbo Blotting System (Bio-Rad). Membrane-bound antibodies were detected by the enhanced chemiluminescence system (Promega). Primary antibodies were the following: rabbit anti-phospho-ERK Thr202/Tyr204 (Cell Signaling, #4376, 1:1000 dilution), rabbit anti-ERK (Cell Signaling Technology #4695, 1:1000 dilution), rabbit anti-phospho-AKT Ser473 (Cell Signaling Technology #4060, 1:2,000 dilution), rabbit anti-AKT (Cell Signaling Technology #4685, 1:1000 dilution), mouse anti-vinculin (Sigma-Aldrich, V9131, 1:2,500 dilution), and mouse anti-α-tubulin (Sigma-Aldrich, T5168, 1:1000 dilution). Images were acquired using the luminescent image analyzer LAS-4000 (Fujifilm). Western blot signal intensities were quantified using ImageJ software.

### Curve fitting of drug sensitivity and synergy studies

For the primary drug screen with single-agent treatments, results were normalized to untreated controls. Dose-response curves and IC_50_ values were generated by nonlinear regression using a four-parameter logistic model with a variable slope, implemented in GraphPad Prism (v9.5). For synergy studies, cell viability assays were performed as described above and results normalized to untreated controls and expressed as percentage of viability. Combination treatment responses were compared to expected outcomes under non-interacting conditions. Synergy scores were computed using the Zero Interaction Potency (ZIP) model [48]. which integrates assumptions from both the Loewe additivity model [49]. and the Bliss independence model [50].

Synergy interactions were classified according to the following thresholds:

- Score < –10: antagonistic interaction
- Score between –10 and +10: additive interaction
- Score > +10: synergistic interaction

Synergy Scores were calculated using an R (v 4.2.0) package, SynergyFinder (v. 3.6.3) [51]. and a pipeline developed and run with snakemake (v5.4.0) [52].

### PDX studies

Tumor implantation and expansion were performed as previously described [53] in 6-week-old male and female NOD/SCID mice (Charles River Laboratories). Mice with established tumors average volume ~200 mm^3^ were randomized using the Laboratory Assistant Suite [48] and treated with the modalities indicated in the figures. Vehicle compositions were the following: 10% DMSO, 40% PEG300, 5% Tween-80, and 45% saline for MRTX1133; 0.5% carboxymethylcellulose, 0.2% Tween 80 for alpelisib and trametinib. Tumor size was evaluated once weekly by caliper measurements, and the approximate volume of the mass was calculated using the formula 4/3π·(*d*/2)^2^·*D*/2, where d is the minor tumor axis and D is the major tumor axis. Mice were treated for up to 8 weeks or until the tumor’s largest diameter reached ≥15 mm. To ensure consistent comparisons across double- and triple-combination groups, results were deemed interpretable when at least four mice reached the prespecified endpoints in at least one MRTX1133-based dual therapy arm. The size of the animal groups was calculated to measure a 50% difference between placebo and treatment groups with a power of 80% and a *P* value of 0.05; sample sizes were similar to those reported in previous publications [28, 54] and conformed to PDX minimal information standards [55]. Operators were not blinded during measurements. In vivo procedures, including animal randomization, and related biobanking data were managed using the Laboratory Assistant Suite [48].

### Immunohistochemistry and morphometric analyses

Tumor specimens were formalin-fixed, paraffin-embedded, and subjected to immunoperoxidase staining with the following antibodies: rabbit anti-phospho-S6 Ser235/236 (Cell Signaling, #2211); rabbit anti-phospho-ERK Thr202/Tyr204 (Cell Signaling, #4370). After incubation with secondary antibodies, immunoreactivities were revealed by incubation in DAB chromogen (Dako). Images were captured with the Leica LAS EZ software using a Leica DM LB microscope. Morphometric quantification was performed with ImageJ software using spectral image segmentation. Software outputs were manually verified by visual inspection of digital images.

### Gene expression analyses

#### RNA extraction and sequencing

To obtain bulk RNA-seq data, RNA was extracted using the Maxwell^®^ RSC miRNA tissue kit (Promega), according to the manufacturer’s protocol, using the Maxwell^®^ Instrument (Promega). The quantification and quality analysis of RNA was performed on a Bioanalyzer 2100 (Agilent), using RNA 6000 Nano Kit (Agilent). Total RNA was processed for RNA-seq analysis with the TruSeq RNA Library Prep Kit v2 (Illumina) following manufacturer’s instructions, and sequenced on a NovaSeq system (Illumina). Single-end 150 bp reads were obtained, aiming at ~50M reads for each sample. Analyses were conducted on three biological replicates for each treatment condition.

#### Quality control, alignment, and expression quantification

Sequencing quality was initially assessed using fastqc (version 0.11.9), and results were summarized with multiqc (version 1.14). Read counts were generated using the analytical pipeline described previously [56], with the exception that sequence alignment was performed exclusively against the human reference genome (GRCh38.p10, GENCODE v27) using STAR (v2.7.1a). All samples yielded more than 40M reads, with ≥ 70% of reads assigned to annotated genes, and therefore met the quality criteria for inclusion in downstream analyses. The computational pipeline used for these analyses is available at: https://github.com/molinerisLab/StromaDistiller.

#### Differential expression analysis

Differential gene expression analysis was performed using DESeq2 (v1.38.3) within the R environment (v4.2.2). The model was specified using the design formula ~ *treat* + *replicate*, where *treat* represents the treatment condition and *replicate* accounts for the three biological replicates. Prior to testing, lowly expressed genes were filtered out by removing those with fewer than 5 reads in more than one sample. Differentially expressed genes (DEGs) were defined using an absolute log₂ fold change ≥ 0.5849625 (corresponding to a ≥1.5-fold change) and an adjusted *P* value < 0.05. The resulting DEG lists were used for GSEA leveraging the R packages clusterProfiler (v4.10.0), DOSE (v3.28.1), msigdbr (v7.5.1), and enrichplot (v1.22.0). The analysis scripts are available at: https://github.com/vodkatad/biodiversa_DE.

### Statistical analyses

The number of biological (nontechnical) replicates for each experiment is reported in the figure legends. Sample sizes were not determined based on a pre-specified effect size. No experimental data points, samples, or animals were excluded from the analyses, and variance was comparable across the groups subjected to statistical comparison. For experiments with two groups, statistical analysis was performed by a two-tailed Welch *t* test. For experiments with more than two groups, one-way ANOVA was used. In case of multiple testing, we adopted the Šídák correction for multiple comparisons. Statistical analyses in the survival experiments were performed by log-rank (Mantel–Cox) test. The level of statistical significance was set at *P* < 0.05. Graphs were generated and statistical analyses were performed using the GraphPad Prism (v9.5) statistical package and R (v3.6.3), including pheatmap (v1.0.12).

### Ethics approval and consent to participate

Tumor samples were obtained from patients undergoing liver metastasectomy at the Candiolo Cancer Institute FPO IRCCS (Candiolo, Torino, Italy) and Ospedale Mauriziano Umberto I (Torino). Written informed consent was obtained from all patients prior to sample collection. Samples were procured and the study was conducted within the institutional study “Prospective study for the determination of the molecular profile of resistance to antineoplastic treatments”—PROFILING protocol No. 001-IRCC-00IIS-10—approved by the Ethics Committee of the Candiolo Cancer Institute FPO IRCCS (authorization v. 11.0, dated 13.07.2022). All studies involving animals were performed in accordance with EU Directive 2010/63/EU and institutional guidelines, regulations, and authorizations. Experimental procedures were reviewed and approved by the Institutional Animal Care and Use Committee (IACUC) of the Candiolo Cancer Institute FPO IRCCS and by the Italian Ministry of Health (authorization 37/2022-PR, dated 25.01.2022), in compliance with all relevant ethical regulations. The maximal tumor size authorized (largest diameter ≥15 mm) was never exceeded in any experiment.

## Supplementary information

Supplementary Figure 1

Uncropped Blots

Supplementary Table 1

Supplementary Table 2

Supplementary Table 3

Supplementary Table 4

Supplementary Table 5

## Data Availability

The RNA-seq data analyzed to evaluate the gene expression levels of EGFR and EGFR ligands (Supplementary Table 2) have been deposited in the European Genome-phenome Archive (EGA) under accession number EGAS00001007024, as part of an independent study [19]. The RNA-seq data analyzed to assess the transcriptional consequences of combination therapies with MRTX1133 have been deposited in EGA under accession number EGAS50000001700. To ensure patient privacy, as required by law, access to the raw data deposited in EGA is controlled by a Data Access Committee (DAC) represented by EG and LT. Researchers may request access through EGA; requests are forwarded to the DAC, which typically responds within approximately 2 weeks and determines the duration of access. The biological samples used in this study (PDXs and tumoroids) are available from the corresponding authors, AB and LT, under a material transfer agreement with the Candiolo Cancer Institute FPO-IRCCS. All original and uncropped western blots are included in the Supplemental Material.
