# Supplementary material for: Efficacy of dual KRASG12D–EGFR blockade versus triple combinations in patient-derived models of KRASG12D-mutant colorectal cancer
Source: Cell Death Dis. 2026 May 28;17(1):657. doi: 10.1038/s41419-026-08900-0 (PMC13402710; doi:10.1038/s41419-026-08900-0)
Supplement: Supplementary file 2 — Uncropped Blots [file 41419_2026_8900_MOESM2_ESM.pdf]

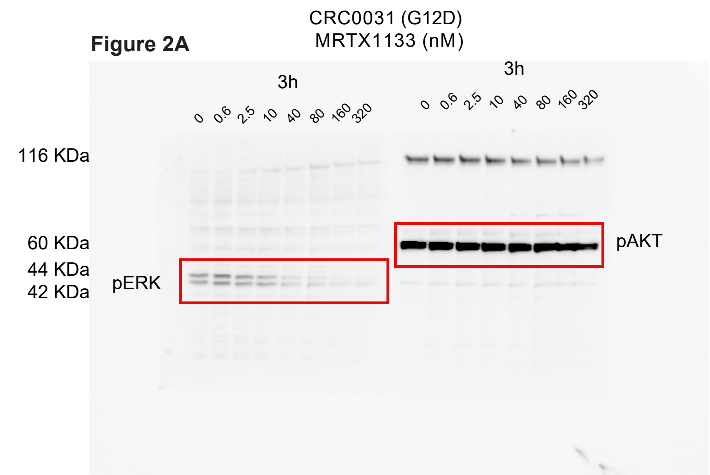
Figure 2A
CRC0031 (G12D)
MRTX1133 (nM)
3h
3h
0 0.6 2.5 10 40 80 160 320
0 0.6 2.5 10 40 80 160 320
116 KDa
60 KDa
44 KDa
42 KDa
pERK
pAKT

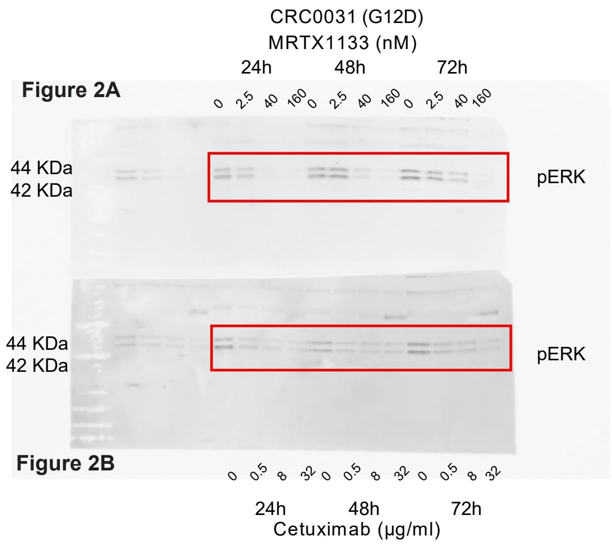
CRC0031 (G12D)
MRTX1133 (nM)
24h 48h 72h
Figure 2A
0 2.5 40 160 0 2.5 40 160 0 2.5 40 160
44 KDa
42 KDa
pERK
44 KDa
42 KDa
pERK
Figure 2B
0 0.5 8 32 0 0.5 8 32 0 0.5 8 32
24h 48h 72h
Cetuximab (μg/ml)

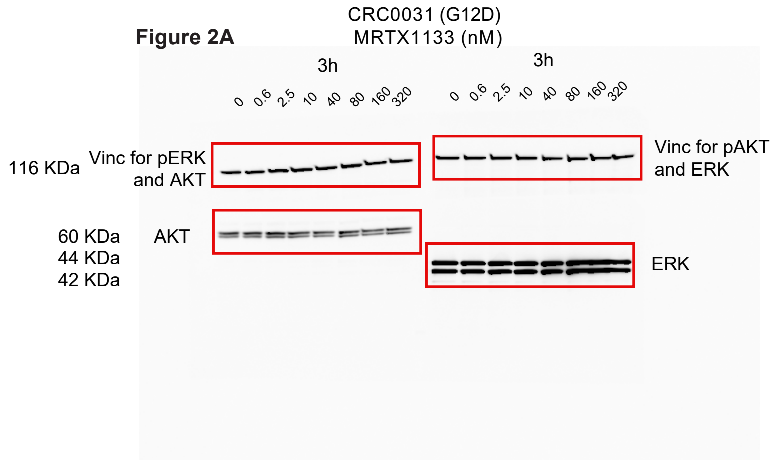
CRC0031 (G12D)
Figure 2A
MRTX1133 (nM)
3h
3h
0 0.6 2.5 10 40 80 160 320
0 0.6 2.5 10 40 80 160 320
116 KDa
Vinc for pERK
and AKT
Vinc for pAKT
and ERK
60 KDa
44 KDa
42 KDa
AKT
ERK

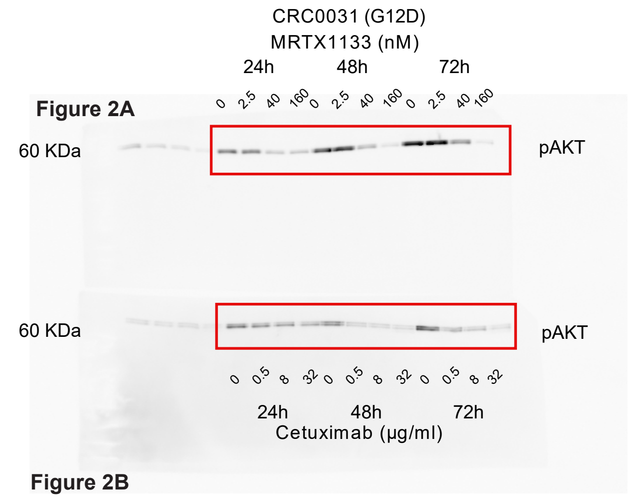
CRC0031 (G12D)
MRTX1133 (nM)
24h 48h 72h
Figure 2A
0 2.5 40 160 0 2.5 40 160 0 2.5 40 160
60 KDa
pAKT
60 KDa
pAKT
0 0.5 8 32 0 0.5 8 32 0 0.5 8 32
24h 48h 72h
Cetuximab (μg/ml)
Figure 2B

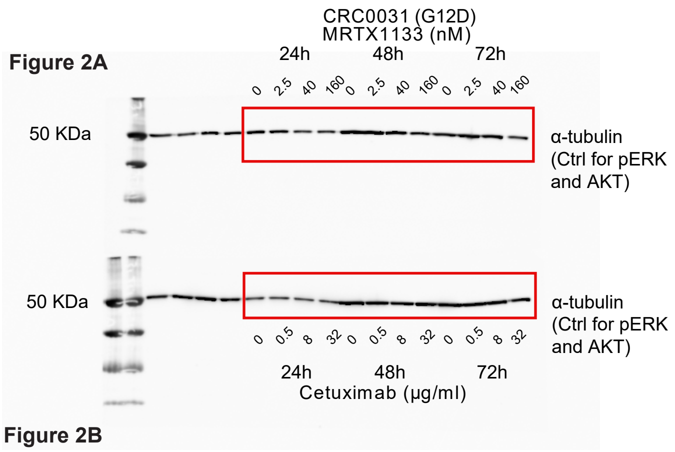
CRC0031 (G12D)
MRTX1133 (nM)
Figure 2A
24h 48h 72h
0 2.5 40 160 0 2.5 40 160 0 2.5 40 160
50 KDa
α-tubulin
(Ctrl for pERK
and AKT)
50 KDa
α-tubulin
(Ctrl for pERK
and AKT)
0 0.5 8 32 0 0.5 8 32 0 0.5 8 32
24h 48h 72h
Cetuximab (μg/ml)
Figure 2B

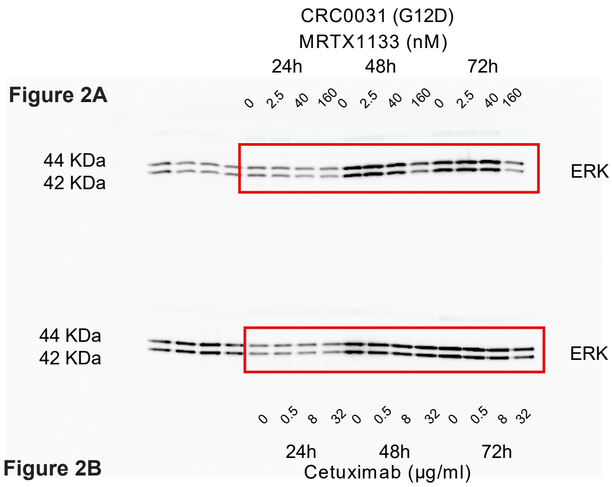
CRC0031 (G12D)
MRTX1133 (nM)
24h 48h 72h
Figure 2A
0 2.5 40 160 0 2.5 40 160 0 2.5 40 160
44 KDa
42 KDa
ERK
44 KDa
42 KDa
ERK
0 0.5 8 32 0 0.5 8 32 0 0.5 8 32
24h 48h 72h
Figure 2B
Cetuximab (μg/ml)

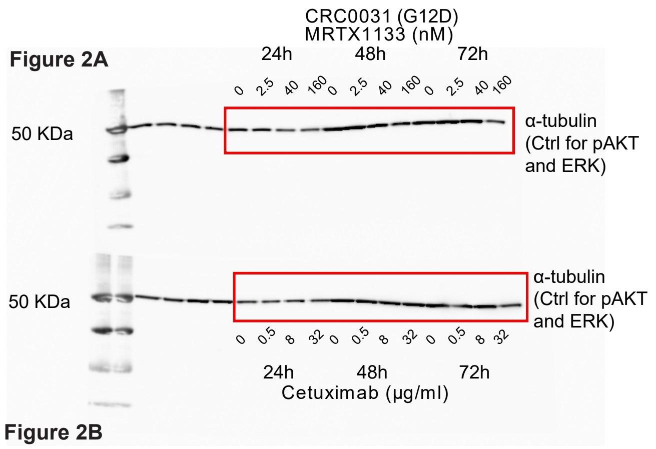
CRC0031 (G12D)
MRTX1133 (nM)
Figure 2A
24h 48h 72h
0 2.5 40 160 0 2.5 40 160 0 2.5 40 160
50 KDa
α-tubulin
(Ctrl for pAKT
and ERK)
50 KDa
α-tubulin
(Ctrl for pAKT
and ERK)
0 0.5 8 32 0 0.5 8 32 0 0.5 8 32
24h 48h 72h
Cetuximab (μg/ml)
Figure 2B

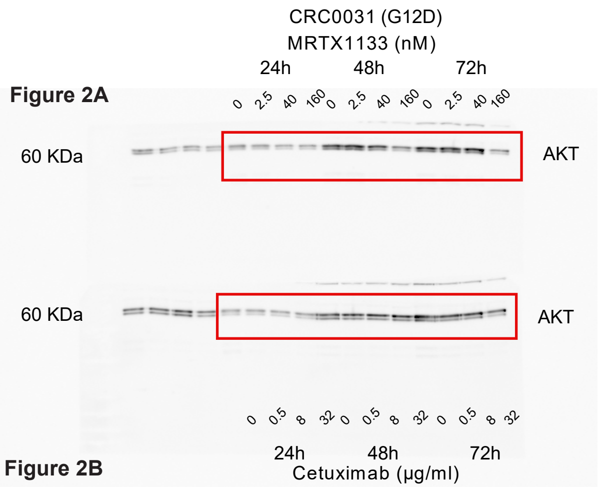
CRC0031 (G12D)
MRTX1133 (nM)
24h 48h 72h
Figure 2A
0 2.5 40 160 0 2.5 40 160 0 2.5 40 160
60 KDa
AKT
60 KDa
AKT
0 0.5 8 32 0 0.5 8 32 0 0.5 8 32
24h 48h 72h
Figure 2B
Cetuximab (μg/ml)

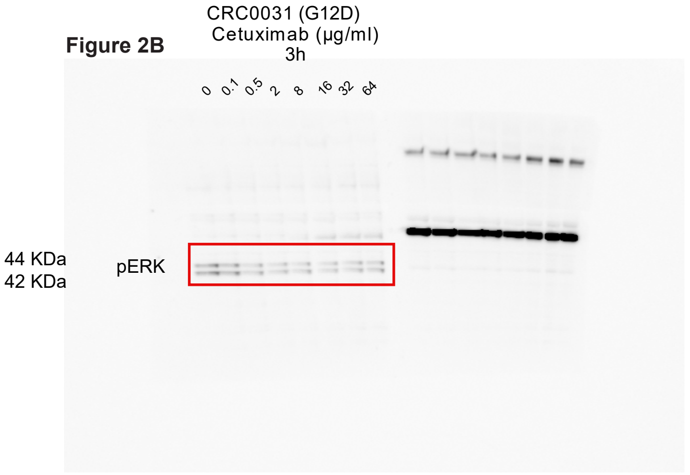

Figure 2B

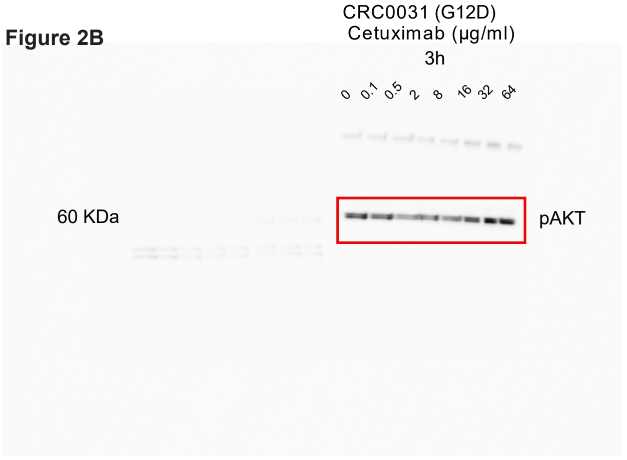

Figure 2B

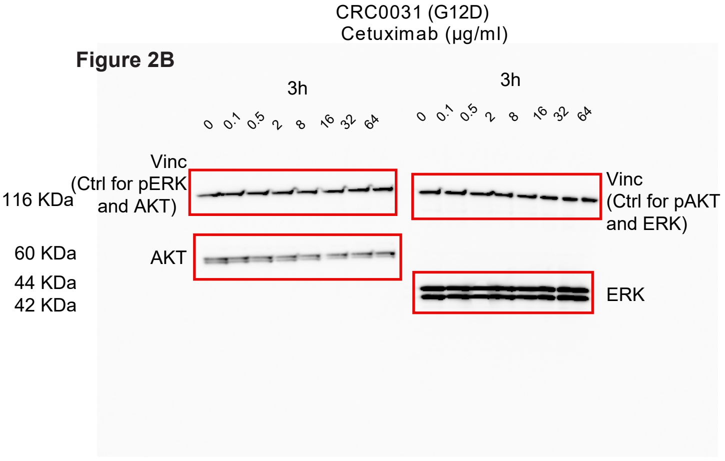

Figure 2B

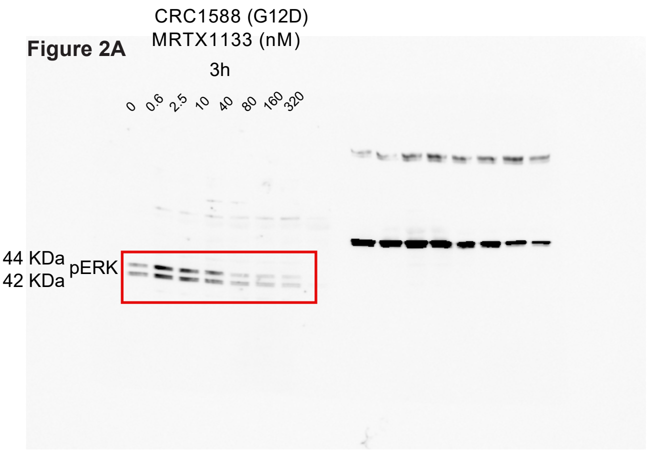

Figure 2A

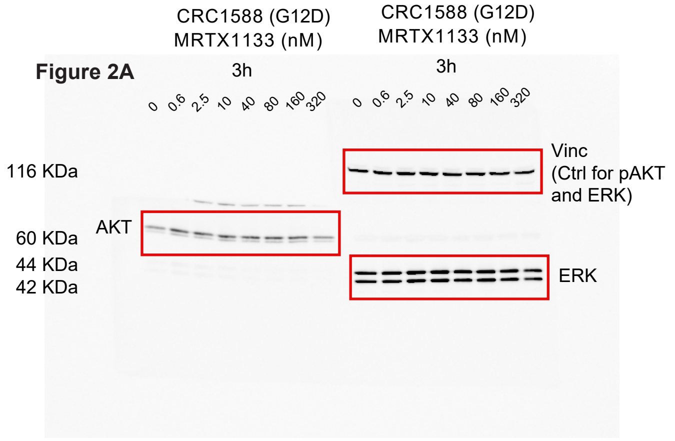

Figure 2A

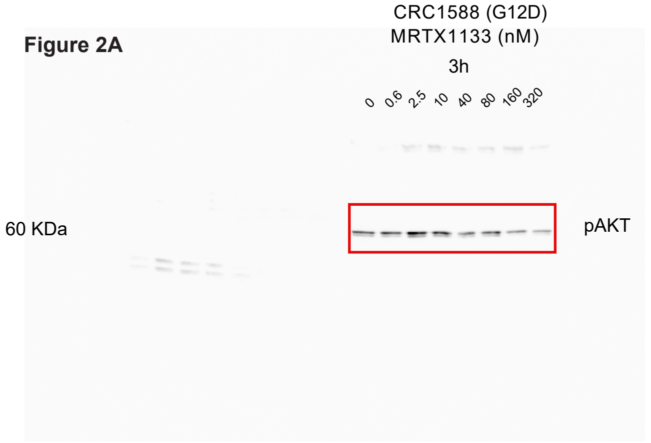

Figure 2A

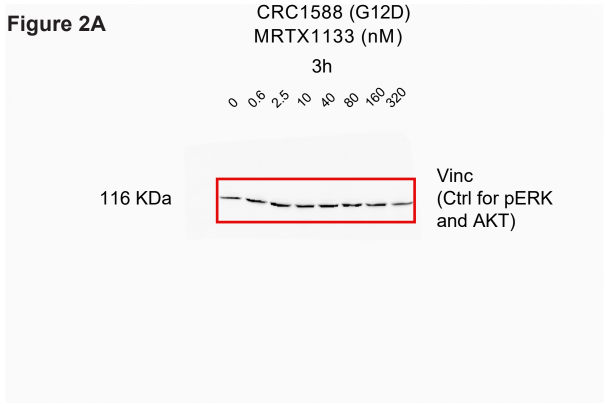

Figure 2A

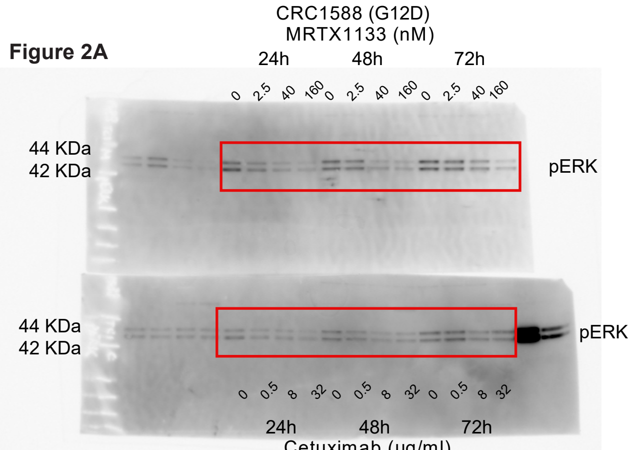

Figure 2A

Figure 2B

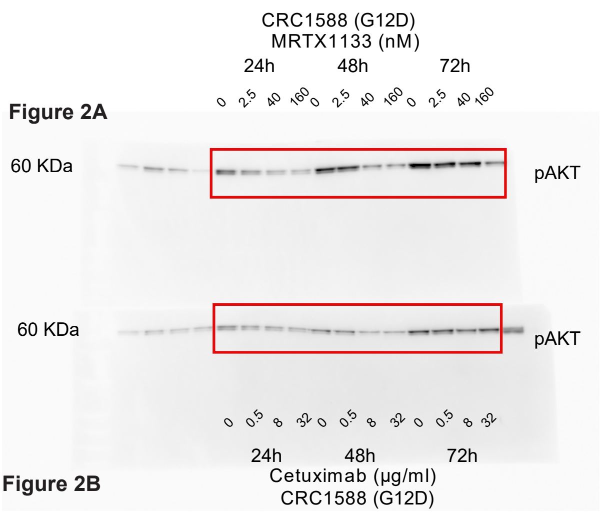
CRC1588 (G12D)
MRTX1133 (nM)
24h 48h 72h
0 2.5 40 160 0 2.5 40 160 0 2.5 40 160
Figure 2A
60 KDa
pAKT
60 KDa
pAKT
0 0.5 8 32 0 0.5 8 32 0 0.5 8 32
24h 48h 72h
Cetuximab (µg/ml)
CRC1588 (G12D)
Figure 2B

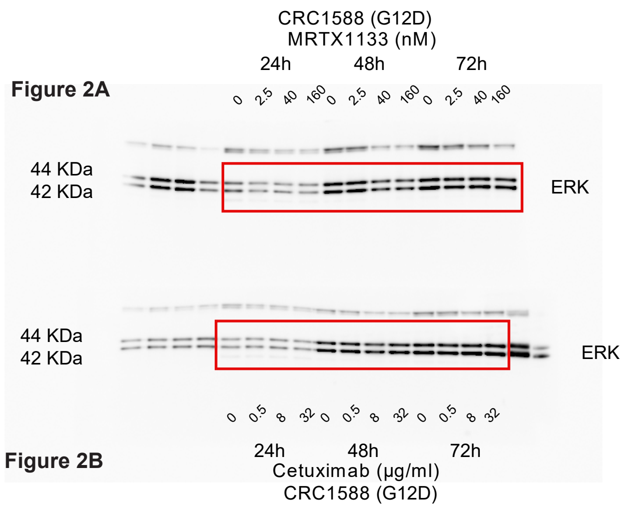
CRC1588 (G12D)
MRTX1133 (nM)
24h 48h 72h
Figure 2A
0 2.5 40 160 0 2.5 40 160 0 2.5 40 160
44 KDa
42 KDa
ERK
44 KDa
42 KDa
ERK
0 0.5 8 32 0 0.5 8 32 0 0.5 8 32
24h 48h 72h
Figure 2B
Cetuximab (µg/ml)
CRC1588 (G12D)

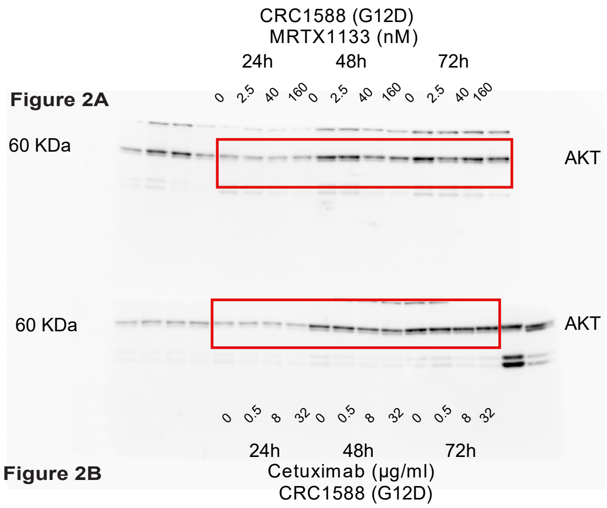
CRC1588 (G12D)
MRTX1133 (nM)
24h 48h 72h
Figure 2A
0 2.5 40 160 0 2.5 40 160 0 2.5 40 160
60 KDa
AKT
60 KDa
AKT
0 0.5 8 32 0 0.5 8 32 0 0.5 8 32
24h 48h 72h
Figure 2B
Cetuximab (µg/ml)
CRC1588 (G12D)

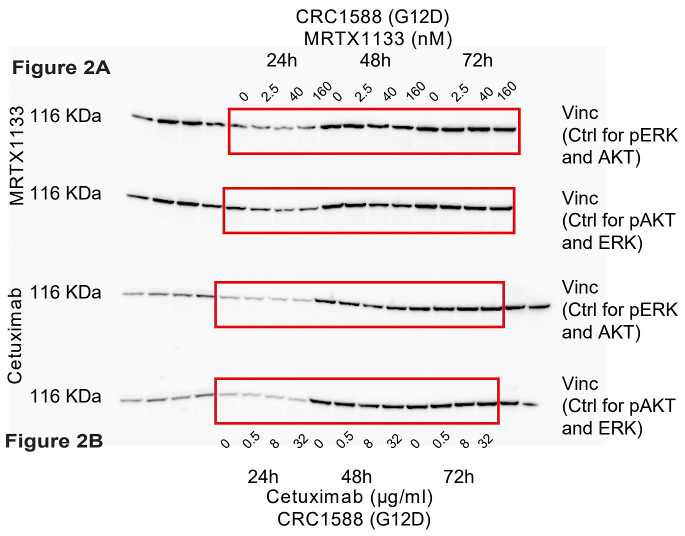
CRC1588 (G12D)
MRTX1133 (nM)
24h 48h 72h
Figure 2A
0 2.5 40 160 0 2.5 40 160 0 2.5 40 160
MRTX1133
116 KDa
Vinc
(Ctrl for pERK
and AKT)
116 KDa
Vinc
(Ctrl for pAKT
and ERK)
Cetuximab
116 KDa
Vinc
(Ctrl for pERK
and AKT)
116 KDa
Vinc
(Ctrl for pAKT
and ERK)
Figure 2B
0 0.5 8 32 0 0.5 8 32 0 0.5 8 32
24h 48h 72h
Cetuximab (µg/ml)
CRC1588 (G12D)

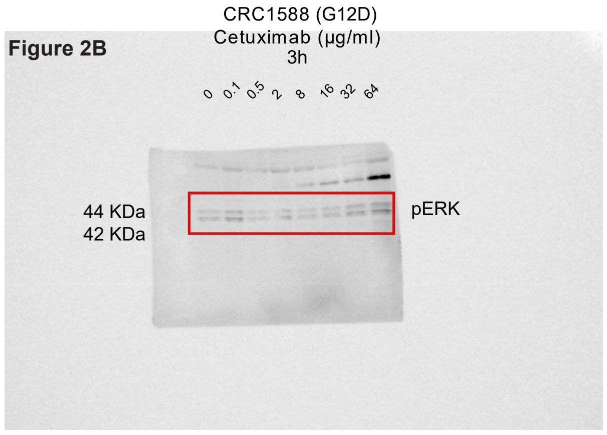
CRC1588 (G12D)
Cetuximab (µg/ml)
3h
Figure 2B
0 0.1 0.5 2 8 16 32 64
44 KDa
42 KDa
pERK

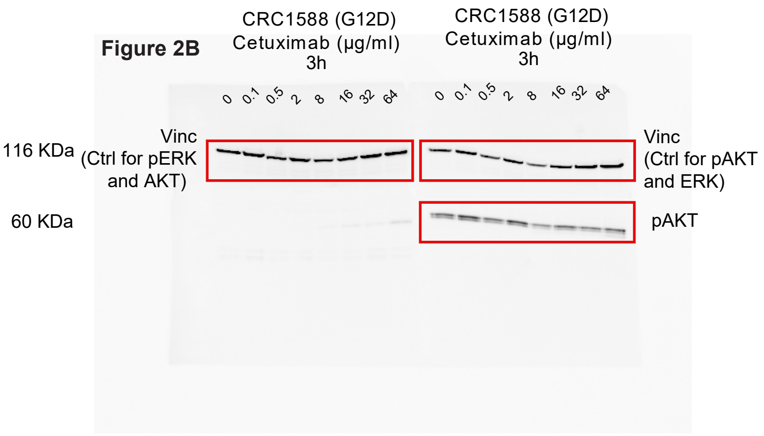
Figure 2B
CRC1588 (G12D)
Cetuximab (µg/ml)
3h
CRC1588 (G12D)
Cetuximab (µg/ml)
3h
0 0.1 0.5 2 8 16 32 64
0 0.1 0.5 2 8 16 32 64
116 KDa
Vinc
(Ctrl for pERK
and AKT)
Vinc
(Ctrl for pAKT
and ERK)
60 KDa
pAKT

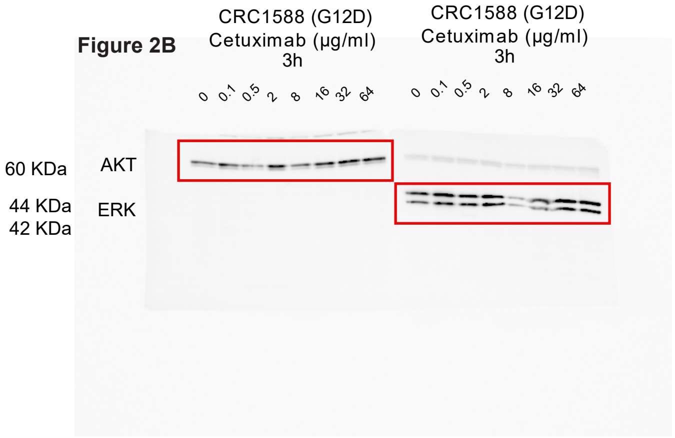
Figure 2B
CRC1588 (G12D)
Cetuximab (µg/ml)
3h
CRC1588 (G12D)
Cetuximab (µg/ml)
3h
0 0.1 0.5 2 8 16 32 64
0 0.1 0.5 2 8 16 32 64
60 KDa
AKT
44 KDa
42 KDa
ERK

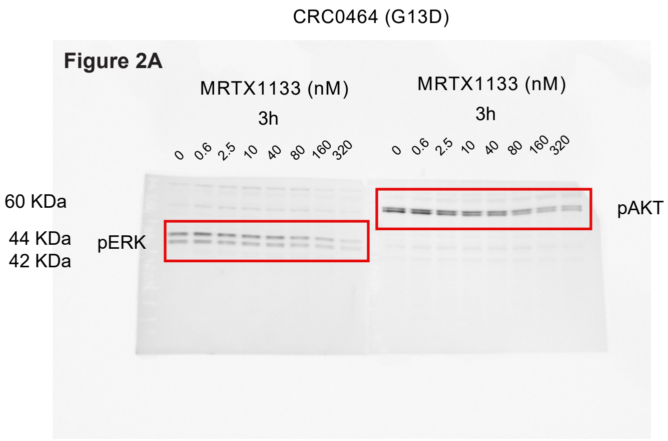
CRC0464 (G13D)
Figure 2A
MRTX1133 (nM)
3h
MRTX1133 (nM)
3h
0 0.6 2.5 10 40 80 160 320
0 0.6 2.5 10 40 80 160 320
60 KDa
pAKT
44 KDa
42 KDa
pERK

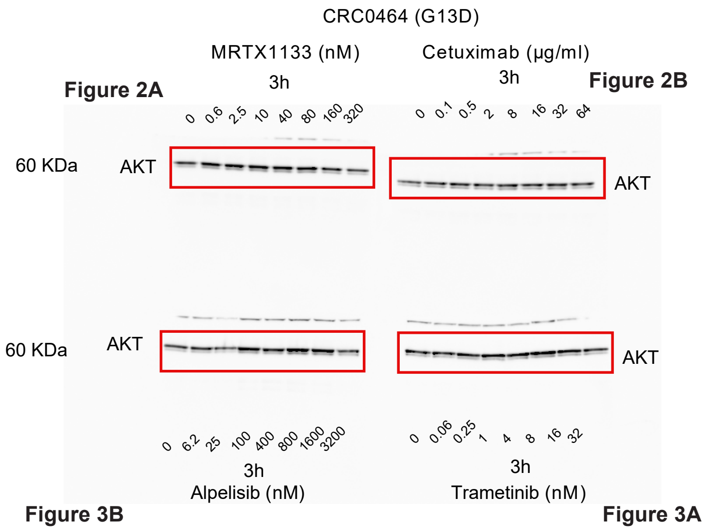
CRC0464 (G13D)
MRTX1133 (nM)
Cetuximab (µg/ml)
3h
3h
Figure 2A
Figure 2B
0 0.6 2.5 10 40 80 160 320
0 0.1 0.5 2 8 16 32 64
60 KDa
AKT
AKT
60 KDa
AKT
AKT
0 6.2 25 100 400 800 1600 3200
0 0.06 0.25 1 4 8 16 32
3h
3h
Alpelisib (nM)
Trametinib (nM)
Figure 3B
Figure 3A

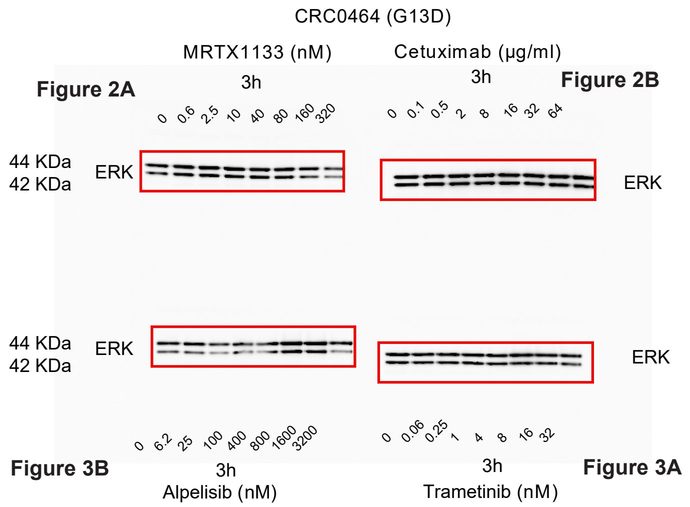
CRC0464 (G13D)
MRTX1133 (nM)
Cetuximab (µg/ml)
3h
3h
Figure 2A
Figure 2B
0 0.6 2.5 10 40 80 160 320
0 0.1 0.5 2 8 16 32 64
44 KDa
42 KDa
ERK
ERK
44 KDa
42 KDa
ERK
ERK
0 6.2 25 100 400 800 1600 3200
0 0.06 0.25 1 4 8 16 32
Figure 3B
3h
3h
Figure 3A
Alpelisib (nM)
Trametinib (nM)

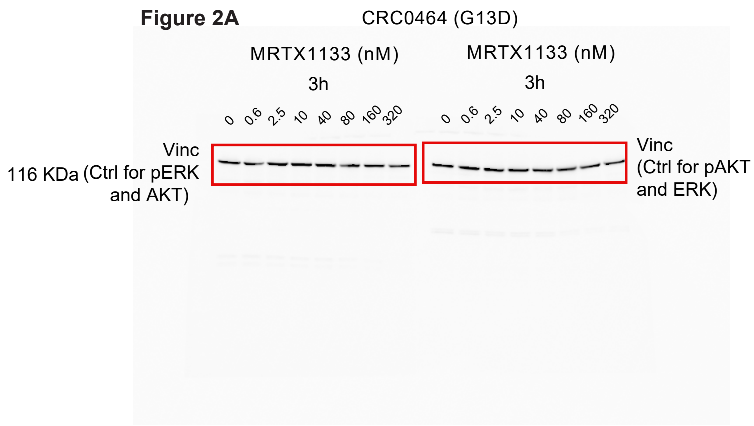
Figure 2A
CRC0464 (G13D)
MRTX1133 (nM)
MRTX1133 (nM)
3h
3h
0 0.6 2.5 10 40 80 160 320
0 0.6 2.5 10 40 80 160 320
Vinc
116 KDa (Ctrl for pERK and AKT)
Vinc
(Ctrl for pAKT and ERK)

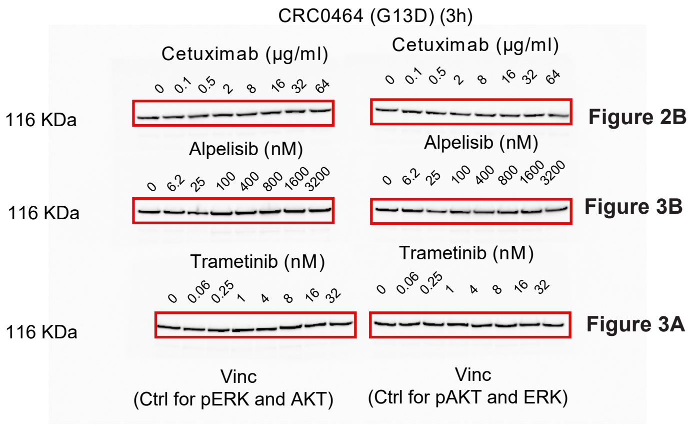
CRC0464 (G13D) (3h)
Cetuximab (µg/ml)
Cetuximab (µg/ml)
0 0.1 0.5 2 8 16 32 64
0 0.1 0.5 2 8 16 32 64
116 KDa
Figure 2B
Alpelisib (nM)
Alpelisib (nM)
0 6.2 25 100 400 800 1600 3200
0 6.2 25 100 400 800 1600 3200
116 KDa
Figure 3B
Trametinib (nM)
Trametinib (nM)
0 0.06 0.25 1 4 8 16 32
0 0.06 0.25 1 4 8 16 32
116 KDa
Figure 3A
Vinc
(Ctrl for pERK and AKT)
Vinc
(Ctrl for pAKT and ERK)

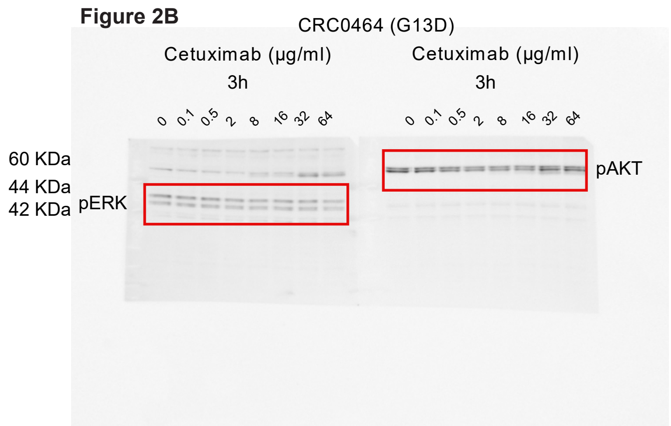
Figure 2B
CRC0464 (G13D)
Cetuximab (µg/ml)
Cetuximab (µg/ml)
3h
3h
0 0.1 0.5 2 8 16 32 64
0 0.1 0.5 2 8 16 32 64
60 KDa
44 KDa
42 KDa
pERK
pAKT

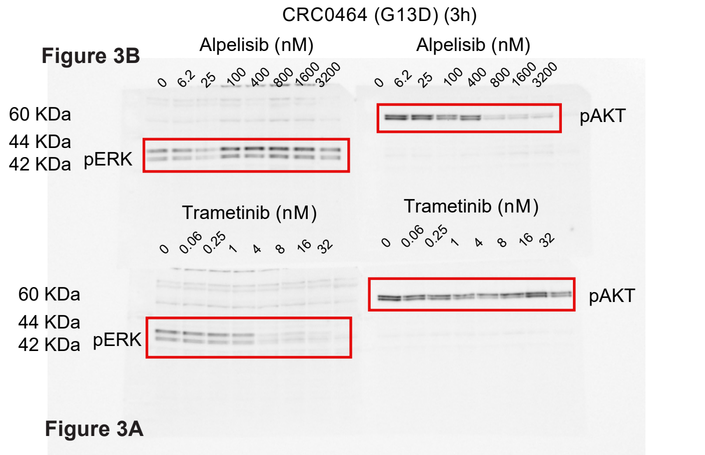
CRC0464 (G13D) (3h)
Figure 3B
Alpelisib (nM)
Alpelisib (nM)
0 6.2 25 100 400 800 1600 3200
0 6.2 25 100 400 800 1600 3200
60 KDa
44 KDa
42 KDa
pERK
pAKT
Trametinib (nM)
Trametinib (nM)
0 0.06 0.25 1 4 8 16 32
0 0.06 0.25 1 4 8 16 32
60 KDa
44 KDa
42 KDa
pERK
pAKT
Figure 3A

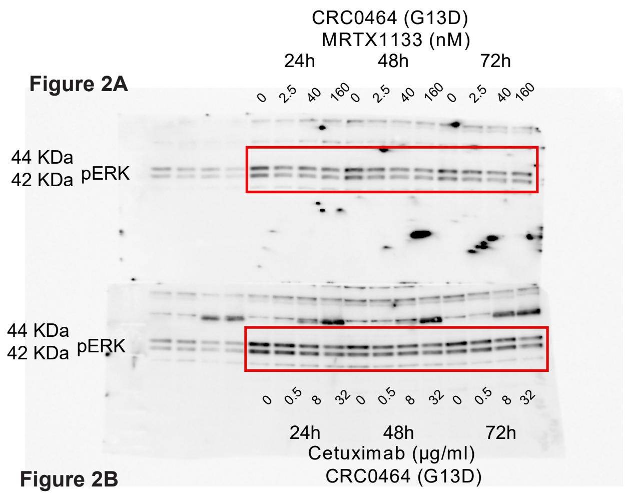
CRC0464 (G13D)
MRTX1133 (nM)
24h 48h 72h
Figure 2A
0 2.5 40 160 0 2.5 40 160 0 2.5 40 160
44 KDa
42 KDa
pERK
44 KDa
42 KDa
pERK
0 0.5 8 32 0 0.5 8 32 0 0.5 8 32
24h 48h 72h
Cetuximab (µg/ml)
CRC0464 (G13D)
Figure 2B

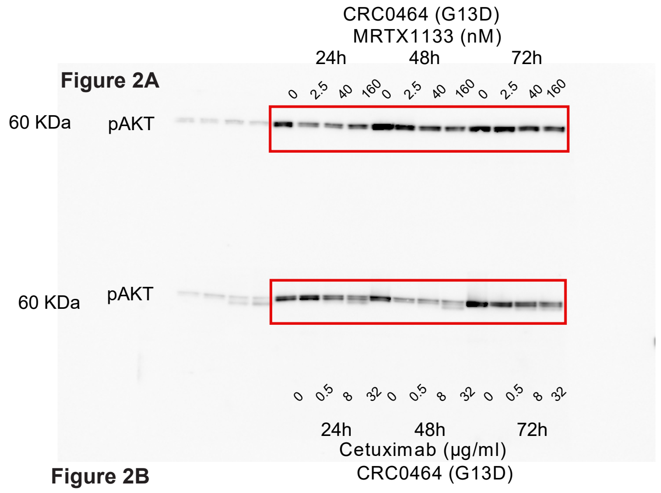
CRC0464 (G13D)
MRTX1133 (nM)
24h 48h 72h
Figure 2A
0 2.5 40 160 0 2.5 40 160 0 2.5 40 160
60 KDa
pAKT
60 KDa
pAKT
0 0.5 8 32 0 0.5 8 32 0 0.5 8 32
24h 48h 72h
Cetuximab (µg/ml)
CRC0464 (G13D)
Figure 2B

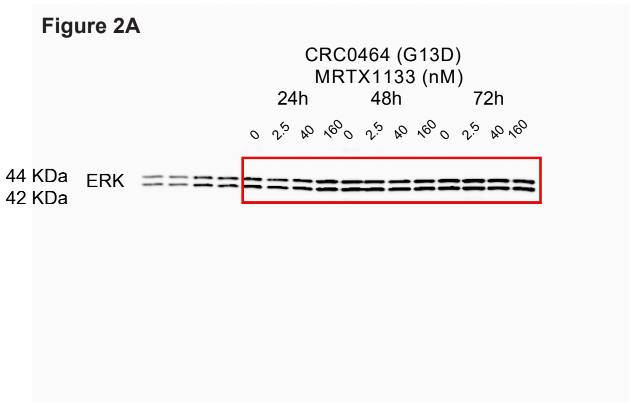
Figure 2A
CRC0464 (G13D)
MRTX1133 (nM)
24h 48h 72h
0 2.5 40 160 0 2.5 40 160 0 2.5 40 160
44 KDa
42 KDa
ERK

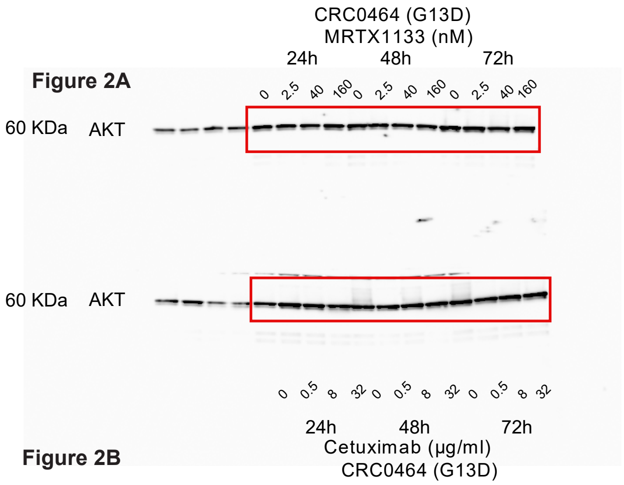
CRC0464 (G13D)
MRTX1133 (nM)
24h 48h 72h
0 2.5 40 160 0 2.5 40 160 0 2.5 40 160
Figure 2A
60 KDa AKT
60 KDa AKT
0 0.5 8 32 0 0.5 8 32 0 0.5 8 32
24h 48h 72h
Cetuximab (µg/ml)
CRC0464 (G13D)
Figure 2B

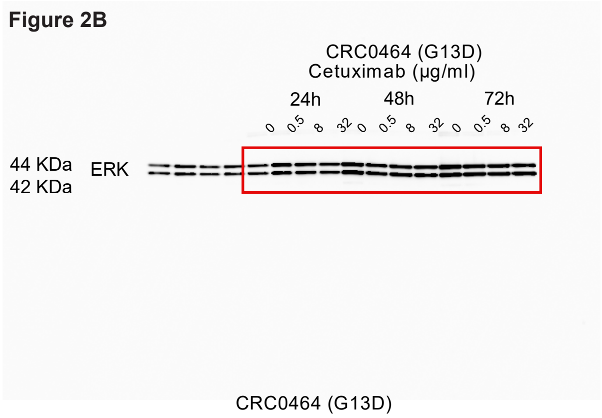
Figure 2B
CRC0464 (G13D)
Cetuximab (µg/ml)
24h 48h 72h
0 0.5 8 32 0 0.5 8 32 0 0.5 8 32
44 KDa
42 KDa
ERK

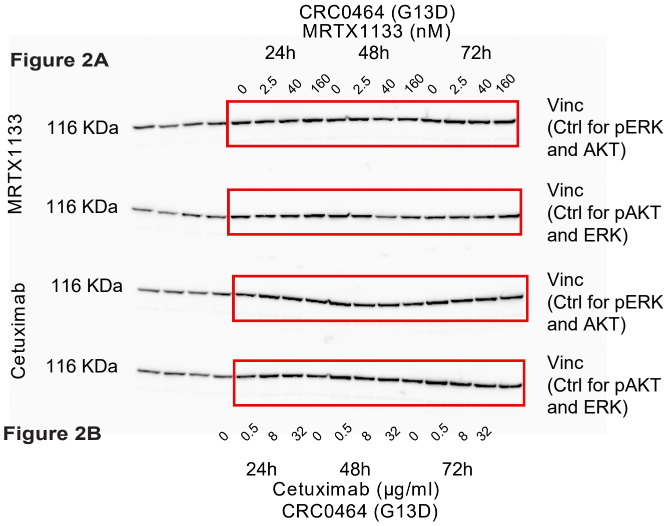
CRC0464 (G13D)
MRTX1133 (nM)
Figure 2A
24h 48h 72h
0 2.5 40 160 0 2.5 40 160 0 2.5 40 160
MRTX1133
116 KDa
Vinc
(Ctrl for pERK
and AKT)
116 KDa
Vinc
(Ctrl for pAKT
and ERK)
Cetuximab
116 KDa
Vinc
(Ctrl for pERK
and AKT)
116 KDa
Vinc
(Ctrl for pAKT
and ERK)
Figure 2B
0 0.5 8 32 0 0.5 8 32 0 0.5 8 32
24h 48h 72h
Cetuximab (µg/ml)
CRC0464 (G13D)

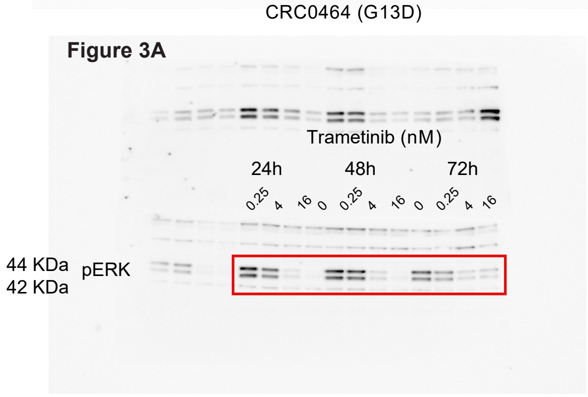
CRC0464 (G13D)
Figure 3A
Trametinib (nM)
24h 48h 72h
0.25 4 16 0 0.25 4 16 0 0.25 4 16
44 KDa
42 KDa
pERK

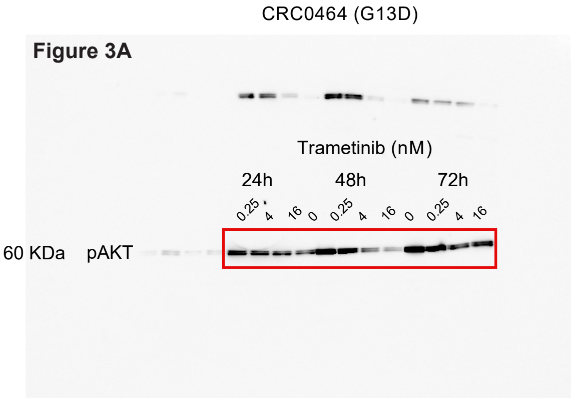
CRC0464 (G13D)
Figure 3A
Trametinib (nM)
24h 48h 72h
0.25 4 16 0 0.25 4 16 0 0.25 4 16
60 KDa pAKT

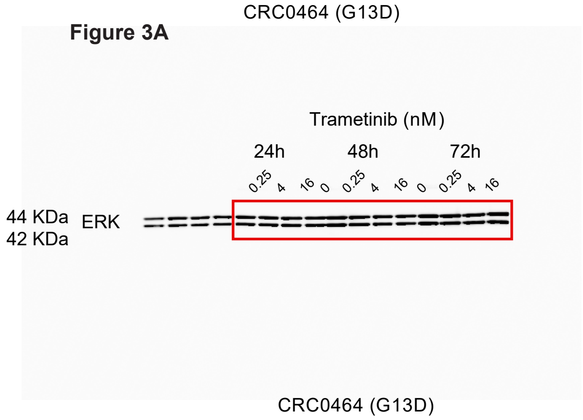
CRC0464 (G13D)
Figure 3A
Trametinib (nM)
24h 48h 72h
0.25 4 16 0 0.25 4 16 0 0.25 4 16
44 KDa
42 KDa
ERK

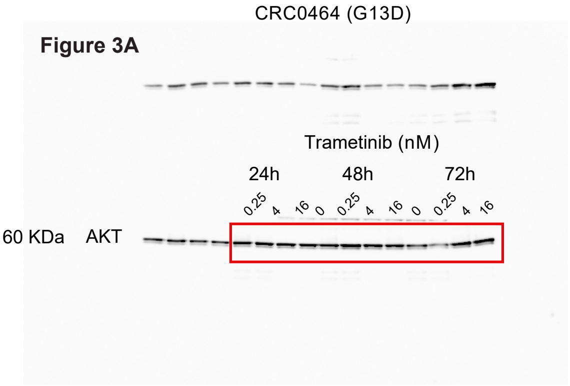
CRC0464 (G13D)
Figure 3A
Trametinib (nM)
24h 48h 72h
0.25 4 16 0 0.25 4 16 0 0.25 4 16
60 KDa AKT

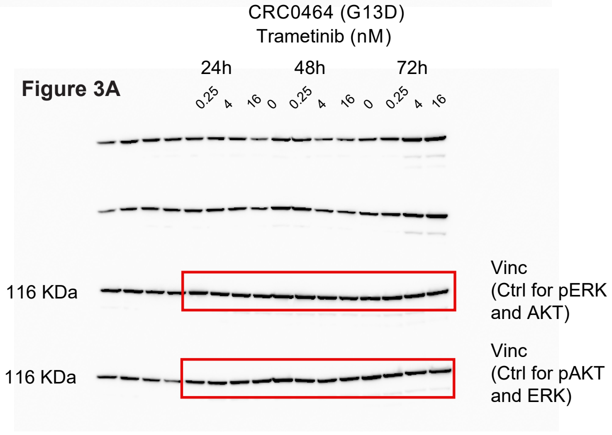
CRC0464 (G13D)
Trametinib (nM)
24h 48h 72h
Figure 3A
0.25 4 16 0 0.25 4 16 0 0.25 4 16
116 KDa
Vinc
(Ctrl for pERK
and AKT)
116 KDa
Vinc
(Ctrl for pAKT
and ERK)

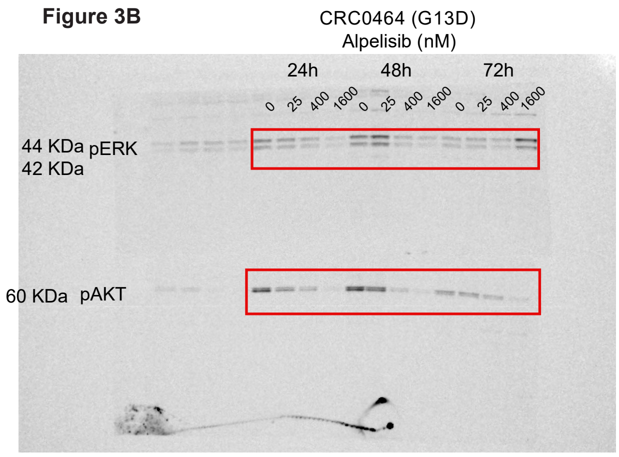
Figure 3B
CRC0464 (G13D)
Alpelisib (nM)
24h
48h
72h
0 25 400 1600 0 25 400 1600 0 25 400 1600
44 KDa pERK
42 KDa
60 KDa pAKT

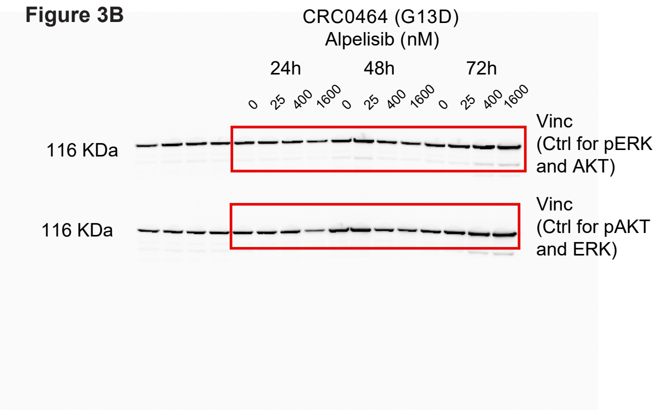
Figure 3B
CRC0464 (G13D)
Alpelisib (nM)
24h
48h
72h
0 25 400 1600 0 25 400 1600 0 25 400 1600
116 KDa
Vinc
(Ctrl for pERK
and AKT)
116 KDa
Vinc
(Ctrl for pAKT
and ERK)

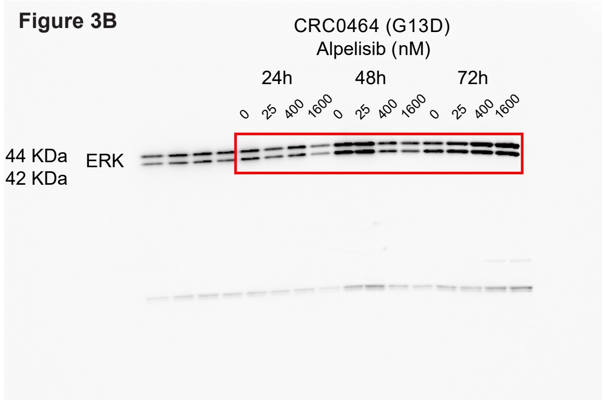
Figure 3B
CRC0464 (G13D)
Alpelisib (nM)
24h
48h
72h
0 25 400 1600 0 25 400 1600 0 25 400 1600
44 KDa ERK
42 KDa

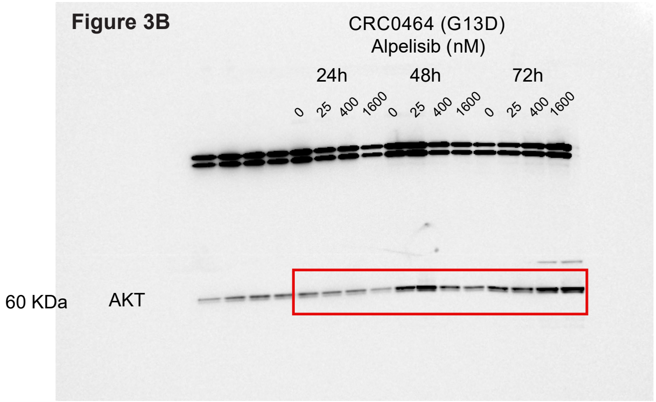
Figure 3B
CRC0464 (G13D)
Alpelisib (nM)
24h
48h
72h
0 25 400 1600 0 25 400 1600 0 25 400 1600
60 KDa AKT

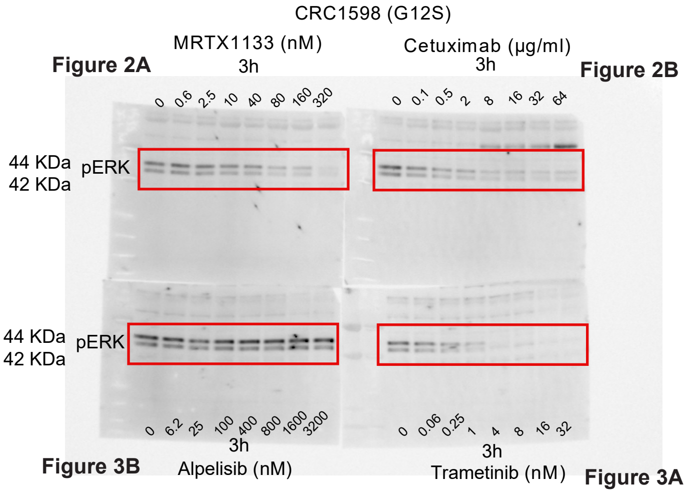
CRC1598 (G12S)
MRTX1133 (nM)
3h
Cetuximab (µg/ml)
3h
Figure 2A
Figure 2B
0 0.6 2.5 10 40 80 160 320
0 0.1 0.5 2 8 16 32 64
44 KDa pERK
42 KDa
44 KDa pERK
42 KDa
0 6.2 25 100 400 800 1600 3200
3h
Alpelisib (nM)
0 0.06 0.25 1 4 8 16 32
3h
Trametinib (nM)
Figure 3B
Figure 3A

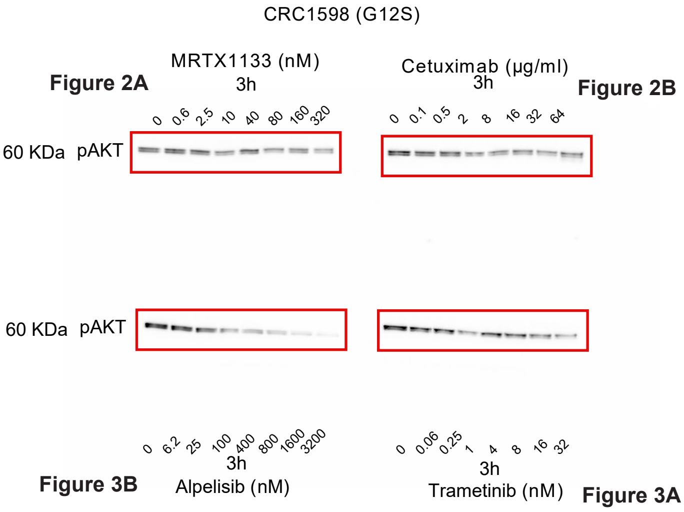
CRC1598 (G12S)
MRTX1133 (nM)
3h
Cetuximab (µg/ml)
3h
Figure 2A
Figure 2B
0 0.6 2.5 10 40 80 160 320
0 0.1 0.5 2 8 16 32 64
60 KDa pAKT
60 KDa pAKT
0 6.2 25 100 400 800 1600 3200
3h
Alpelisib (nM)
0 0.06 0.25 1 4 8 16 32
3h
Trametinib (nM)
Figure 3B
Figure 3A

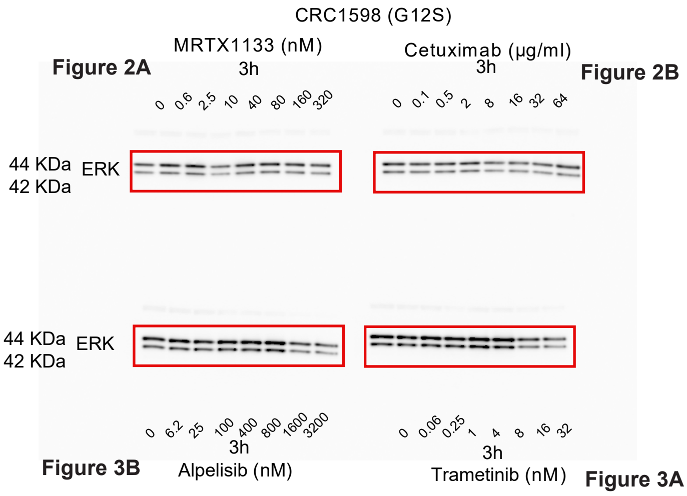
CRC1598 (G12S)
MRTX1133 (nM)
3h
Cetuximab (µg/ml)
3h
Figure 2A
Figure 2B
0 0.6 2.5 10 40 80 160 320
0 0.1 0.5 2 8 16 32 64
44 KDa ERK
42 KDa
44 KDa ERK
42 KDa
0 6.2 25 100 400 800 1600 3200
3h
Alpelisib (nM)
0 0.06 0.25 1 4 8 16 32
3h
Trametinib (nM)
Figure 3B
Figure 3A

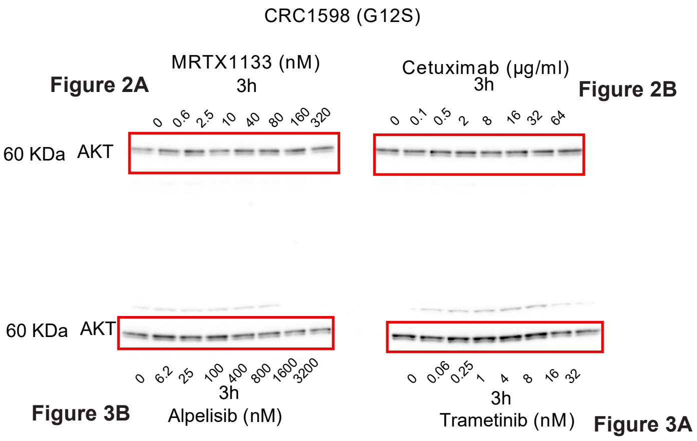
CRC1598 (G12S)
MRTX1133 (nM)
3h
Cetuximab (µg/ml)
3h
Figure 2A
Figure 2B
0 0.6 2.5 10 40 80 160 320
0 0.1 0.5 2 8 16 32 64
60 KDa AKT
60 KDa AKT
0 6.2 25 100 400 800 1600 3200
3h
Alpelisib (nM)
0 0.06 0.25 1 4 8 16 32
3h
Trametinib (nM)
Figure 3B
Figure 3A

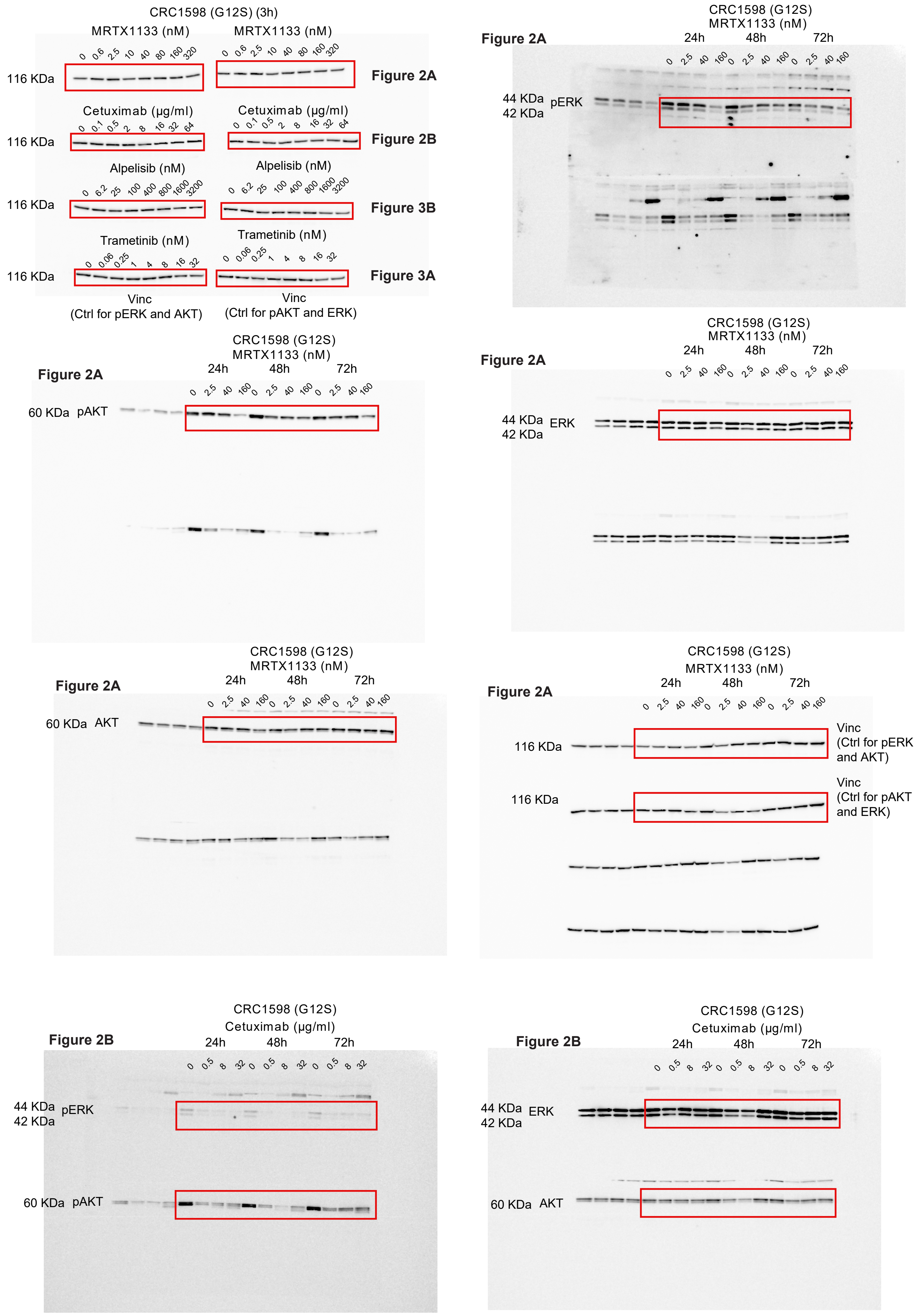
CRC1598 (G12S) (3h)
MRTX1133 (nM)
0 0.6 2.5 10 40 80 160 320
116 KDa
Figure 2A
Cetuximab (μg/ml)
0 0.1 0.5 2 8 16 32 64
Figure 2B
Alpelisib (nM)
0 6.2 25 100 400 800 1600 3200
Figure 3B
Trametinib (nM)
0 0.06 0.25 1 4 8 16 32
Figure 3A
Vinc
(Ctrl for pERK and AKT)
Vinc
(Ctrl for pAKT and ERK)
CRC1598 (G12S)
MRTX1133 (nM)
24h 48h 72h
0 2.5 40 160
44 KDa
42 KDa
pERK
ERK
60 KDa
pAKT
AKT
Vinc
(Ctrl for pERK and AKT)
Vinc
(Ctrl for pAKT and ERK)
Cetuximab (μg/ml)
0 0.5 8 32

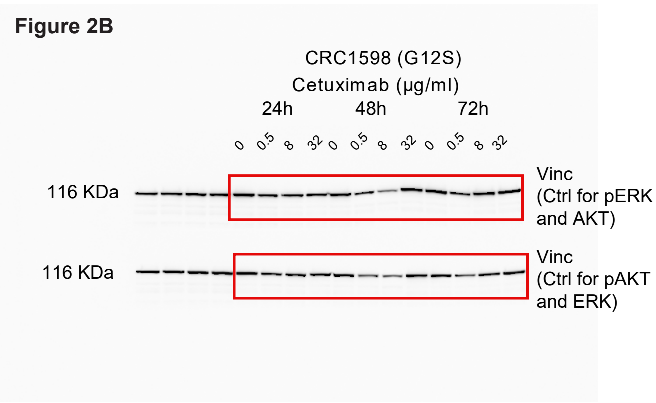
Figure 2B
CRC1598 (G12S)
Cetuximab (µg/ml)
24h 48h 72h
0 0.5 8 32 0 0.5 8 32 0 0.5 8 32
116 KDa
Vinc
(Ctrl for pERK
and AKT)
116 KDa
Vinc
(Ctrl for pAKT
and ERK)

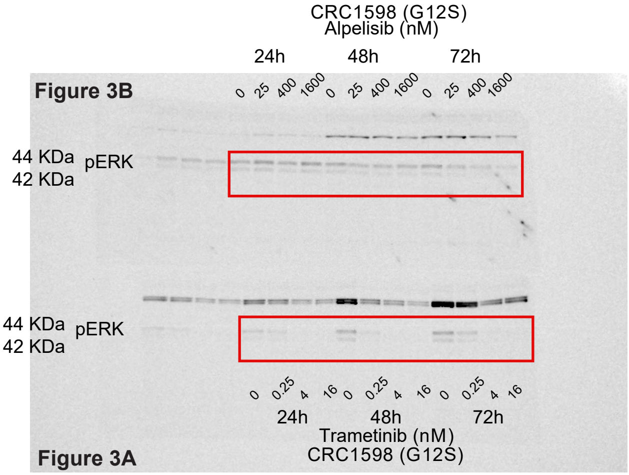
CRC1598 (G12S)
Alpelisib (nM)
24h 48h 72h
Figure 3B
0 25 400 1600 0 25 400 1600 0 25 400 1600
44 KDa
42 KDa
pERK
44 KDa
42 KDa
pERK
0 0.25 4 16 0 0.25 4 16 0 0.25 4 16
24h 48h 72h
Trametinib (nM)
Figure 3A
CRC1598 (G12S)

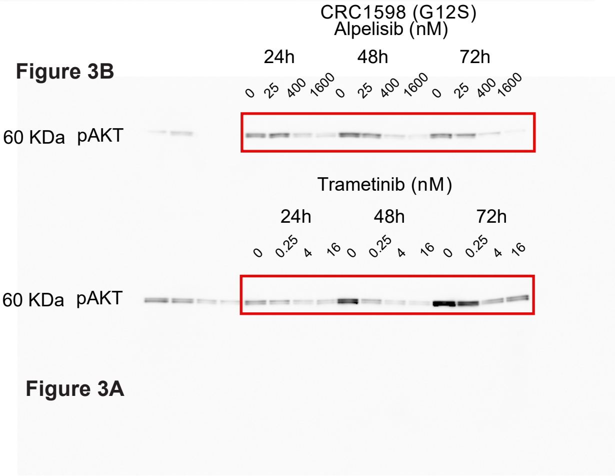
CRC1598 (G12S)
Alpelisib (nM)
Figure 3B
24h 48h 72h
0 25 400 1600 0 25 400 1600 0 25 400 1600
60 KDa pAKT
Trametinib (nM)
24h 48h 72h
0 0.25 4 16 0 0.25 4 16 0 0.25 4 16
60 KDa pAKT
Figure 3A

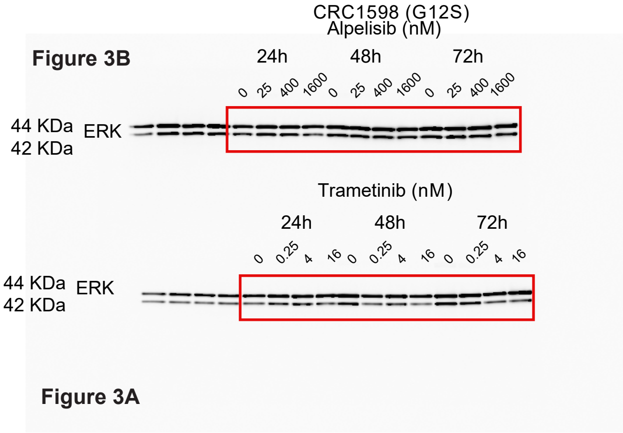
CRC1598 (G12S)
Alpelisib (nM)
Figure 3B
24h 48h 72h
0 25 400 1600 0 25 400 1600 0 25 400 1600
44 KDa
42 KDa
ERK
Trametinib (nM)
24h 48h 72h
0 0.25 4 16 0 0.25 4 16 0 0.25 4 16
44 KDa
42 KDa
ERK
Figure 3A

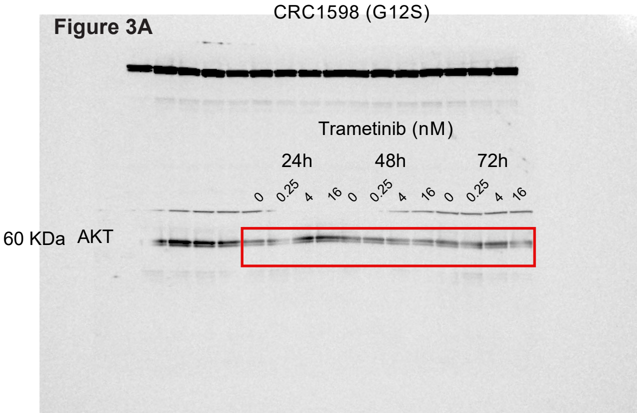
Figure 3A
CRC1598 (G12S)
Trametinib (nM)
24h 48h 72h
0 0.25 4 16 0 0.25 4 16 0 0.25 4 16
60 KDa AKT

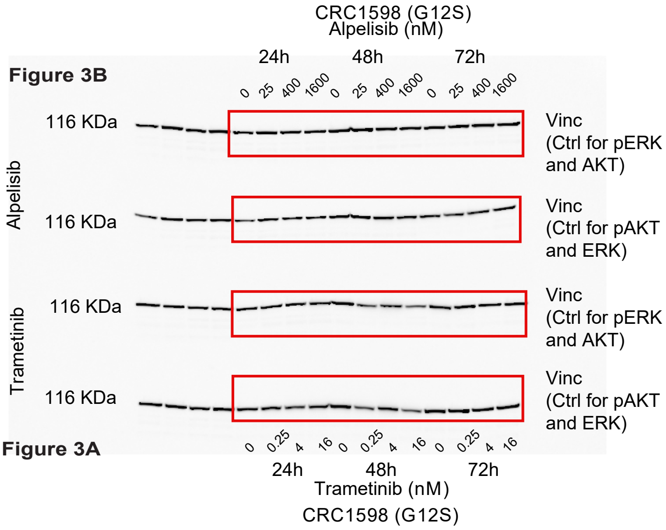
CRC1598 (G12S)
Alpelisib (nM)
24h 48h 72h
Figure 3B
0 25 400 1600 0 25 400 1600 0 25 400 1600
116 KDa
Vinc
(Ctrl for pERK
and AKT)
Alpelisib
116 KDa
Vinc
(Ctrl for pAKT
and ERK)
116 KDa
Vinc
(Ctrl for pERK
and AKT)
Trametinib
116 KDa
Vinc
(Ctrl for pAKT
and ERK)
Figure 3A
0 0.25 4 16 0 0.25 4 16 0 0.25 4 16
24h 48h 72h
Trametinib (nM)
CRC1598 (G12S)

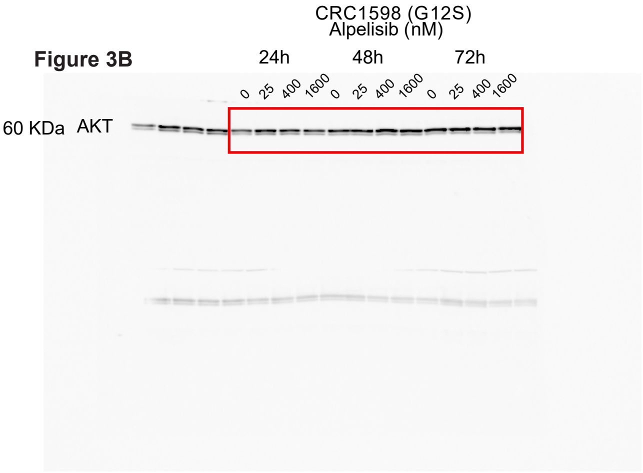
CRC1598 (G12S)
Alpelisib (nM)
Figure 3B
24h 48h 72h
0 25 400 1600 0 25 400 1600 0 25 400 1600
60 KDa AKT

Figure 3A

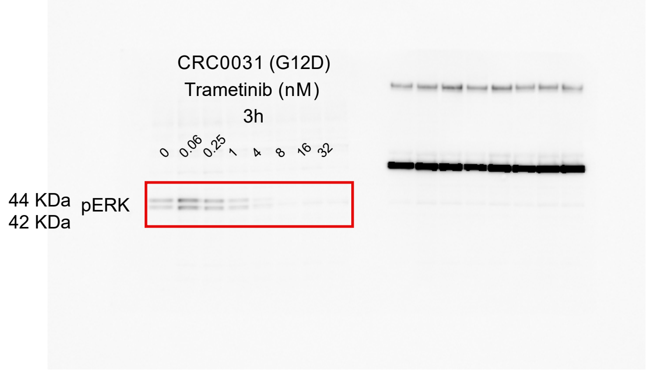

Figure 3A

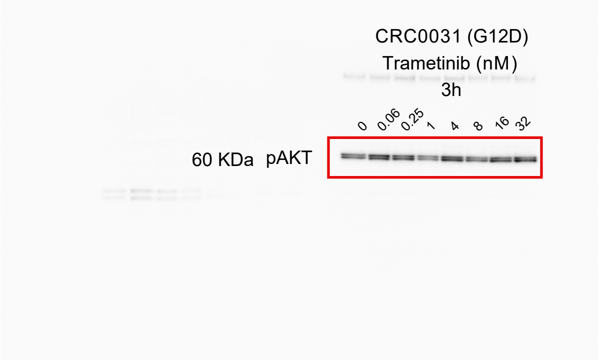

Figure 3A

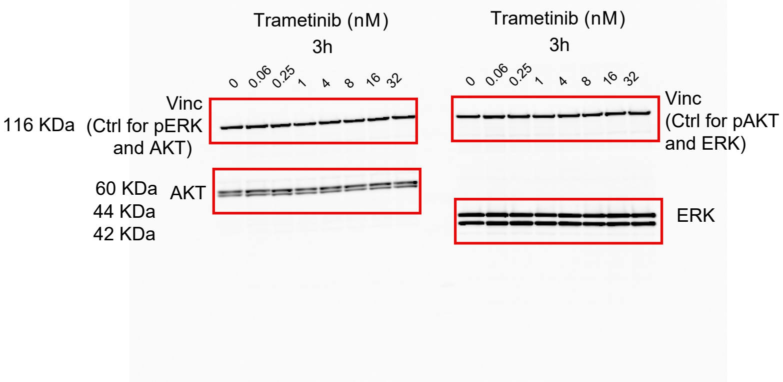

Figure 3B

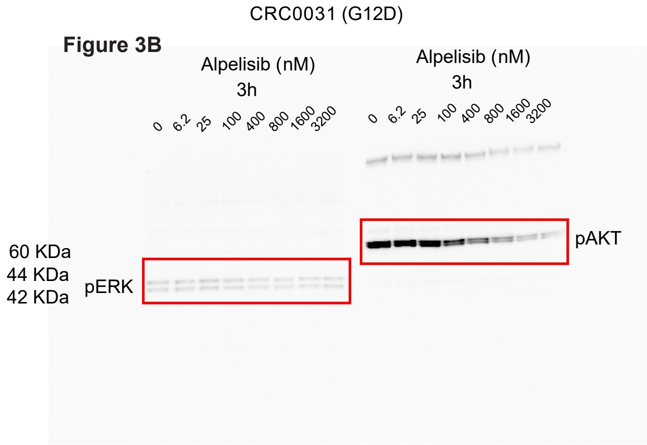

Figure 3B

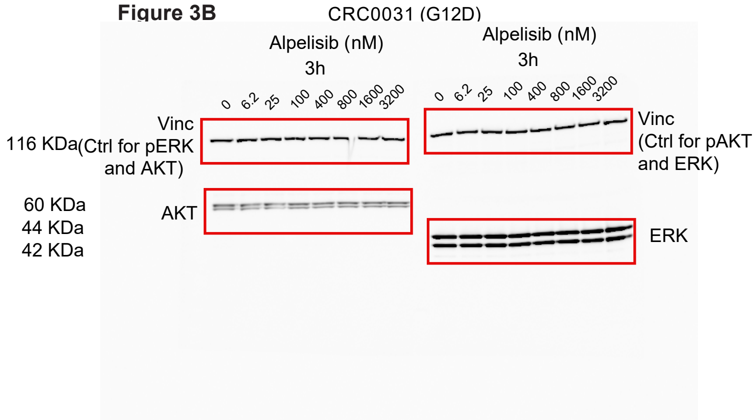

Figure 3B

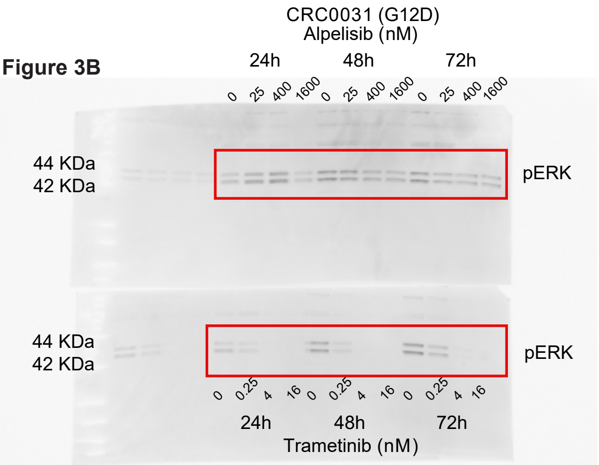

Figure 3A

Figure 3B

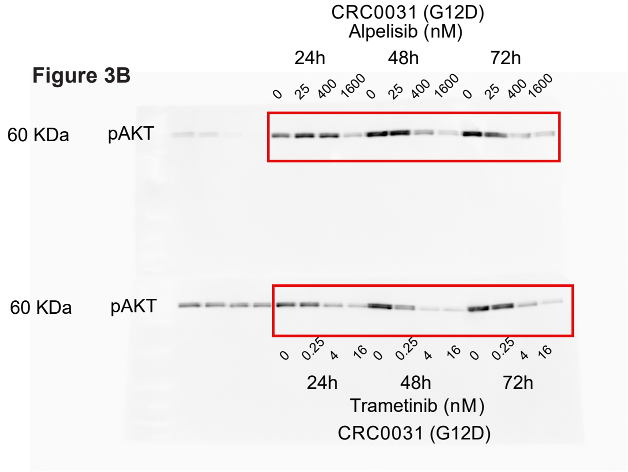

Figure 3A

Figure 3A

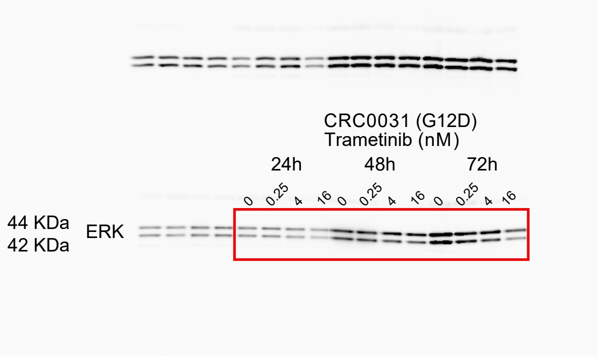

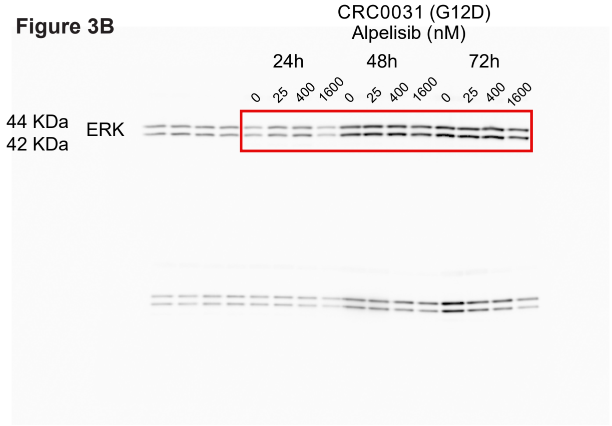
Figure 3B
CRC0031 (G12D)
Alpelisib (nM)
24h 48h 72h
0 25 400 1600 0 25 400 1600 0 25 400 1600
44 KDa
42 KDa
ERK

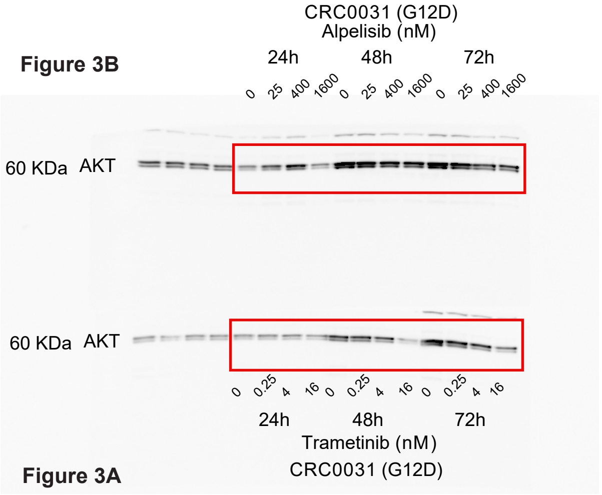
Figure 3B
CRC0031 (G12D)
Alpelisib (nM)
24h 48h 72h
0 25 400 1600 0 25 400 1600 0 25 400 1600
60 KDa AKT
60 KDa AKT
0 0.25 4 16 0 0.25 4 16 0 0.25 4 16
24h 48h 72h
Trametinib (nM)
CRC0031 (G12D)
Figure 3A

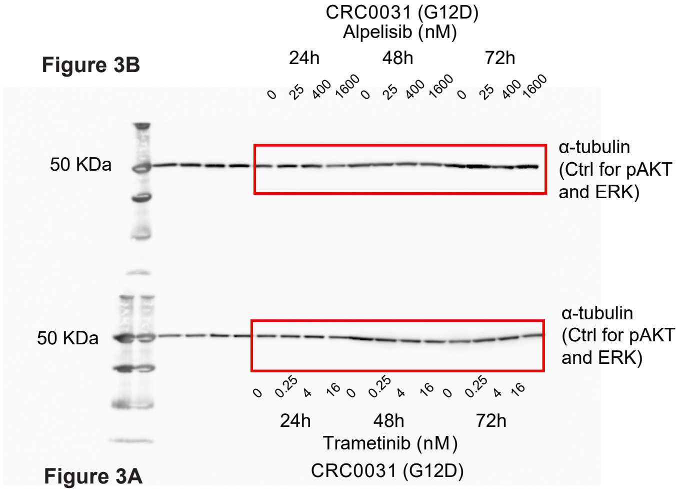
Figure 3B
CRC0031 (G12D)
Alpelisib (nM)
24h 48h 72h
0 25 400 1600 0 25 400 1600 0 25 400 1600
50 KDa
α-tubulin
(Ctrl for pAKT
and ERK)
50 KDa
α-tubulin
(Ctrl for pAKT
and ERK)
0 0.25 4 16 0 0.25 4 16 0 0.25 4 16
24h 48h 72h
Trametinib (nM)
CRC0031 (G12D)
Figure 3A

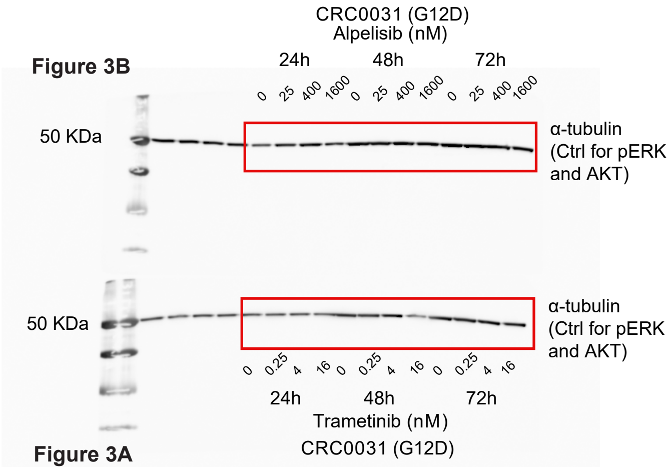
Figure 3B
CRC0031 (G12D)
Alpelisib (nM)
24h 48h 72h
0 25 400 1600 0 25 400 1600 0 25 400 1600
50 KDa
α-tubulin
(Ctrl for pERK
and AKT)
50 KDa
α-tubulin
(Ctrl for pERK
and AKT)
0 0.25 4 16 0 0.25 4 16 0 0.25 4 16
24h 48h 72h
Trametinib (nM)
CRC0031 (G12D)
Figure 3A

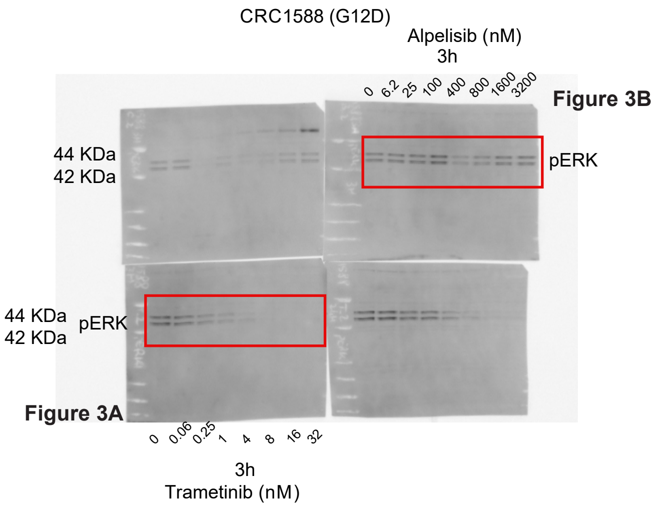
CRC1588 (G12D)
Alpelisib (nM)
3h
0 6.2 25 100 400 800 1600 3200
Figure 3B
44 KDa
42 KDa
pERK
44 KDa
42 KDa
pERK
Figure 3A
0 0.06 0.25 1 4 8 16 32
3h
Trametinib (nM)

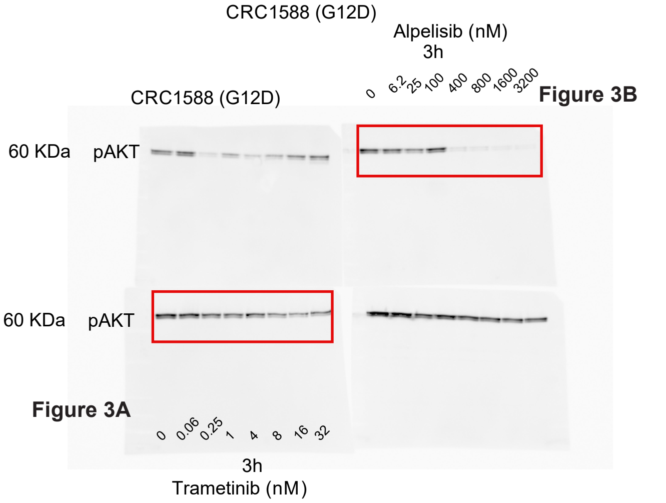
CRC1588 (G12D)
Alpelisib (nM)
3h
0 6.2 25 100 400 800 1600 3200
Figure 3B
CRC1588 (G12D)
60 KDa pAKT
60 KDa pAKT
Figure 3A
0 0.06 0.25 1 4 8 16 32
3h
Trametinib (nM)

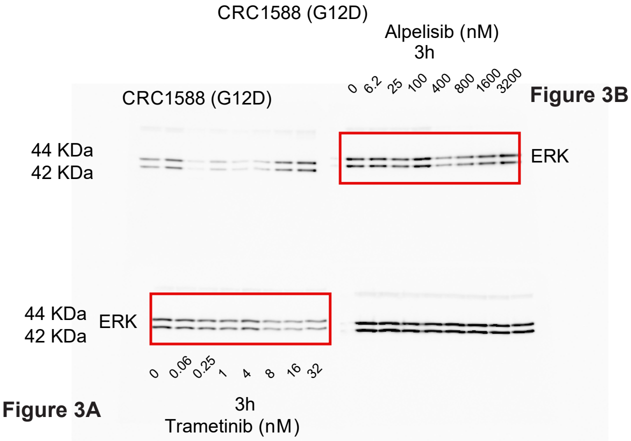
CRC1588 (G12D)
Alpelisib (nM)
3h
0 6.2 25 100 400 800 1600 3200
Figure 3B
CRC1588 (G12D)
44 KDa
42 KDa
ERK
44 KDa
42 KDa
ERK
0 0.06 0.25 1 4 8 16 32
Figure 3A
3h
Trametinib (nM)

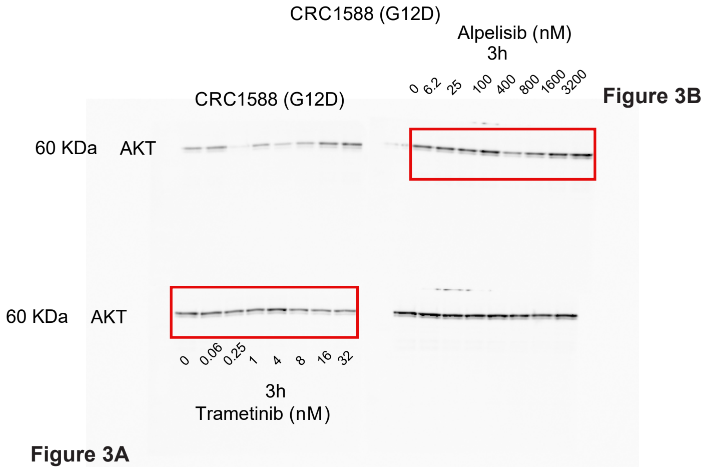
CRC1588 (G12D)
Alpelisib (nM)
3h
0 6.2 25 100 400 800 1600 3200
Figure 3B
CRC1588 (G12D)
60 KDa AKT
60 KDa AKT
0 0.06 0.25 1 4 8 16 32
3h
Trametinib (nM)
Figure 3A

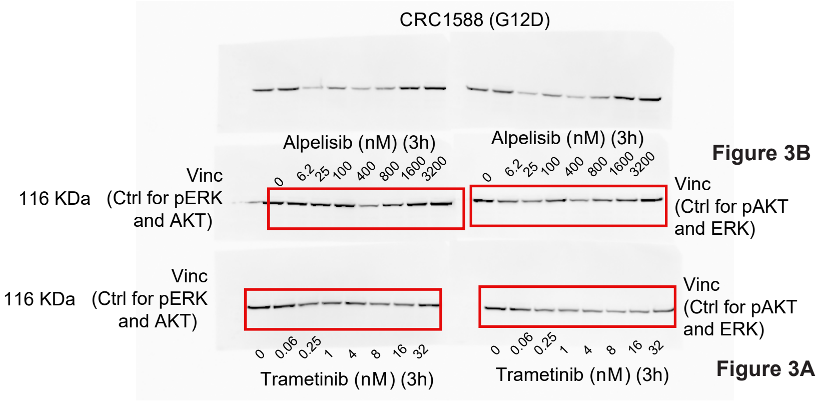
CRC1588 (G12D)
Alpelisib (nM) (3h)
0 6.2 25 100 400 800 1600 3200
Alpelisib (nM) (3h)
0 6.2 25 100 400 800 1600 3200
Figure 3B
Vinc
116 KDa (Ctrl for pERK and AKT)
Vinc (Ctrl for pAKT and ERK)
Vinc
116 KDa (Ctrl for pERK and AKT)
Vinc (Ctrl for pAKT and ERK)
0 0.06 0.25 1 4 8 16 32
Trametinib (nM) (3h)
0 0.06 0.25 1 4 8 16 32
Trametinib (nM) (3h)
Figure 3A

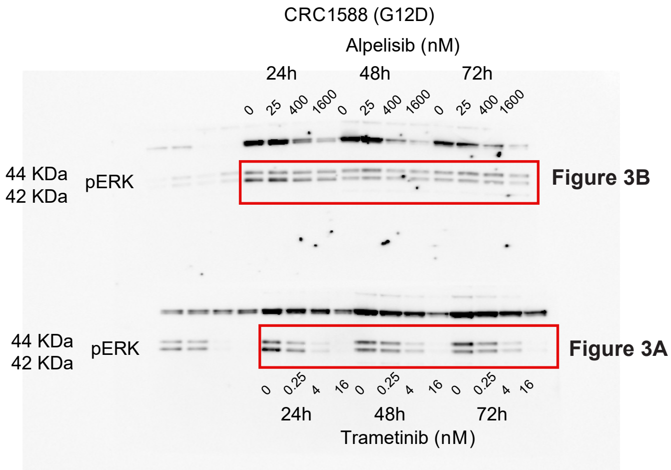
CRC1588 (G12D)
Alpelisib (nM)
24h 48h 72h
0 25 400 1600 0 25 400 1600 0 25 400 1600
44 KDa
42 KDa
pERK
Figure 3B
44 KDa
42 KDa
pERK
Figure 3A
0 0.25 4 16 0 0.25 4 16 0 0.25 4 16
24h 48h 72h
Trametinib (nM)

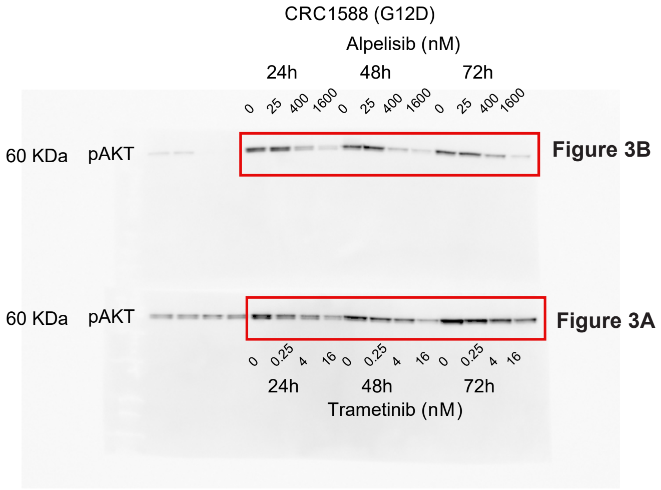
CRC1588 (G12D)
Alpelisib (nM)
24h 48h 72h
0 25 400 1600 0 25 400 1600 0 25 400 1600
60 KDa pAKT
Figure 3B
60 KDa pAKT
Figure 3A
0 0.25 4 16 0 0.25 4 16 0 0.25 4 16
24h 48h 72h
Trametinib (nM)

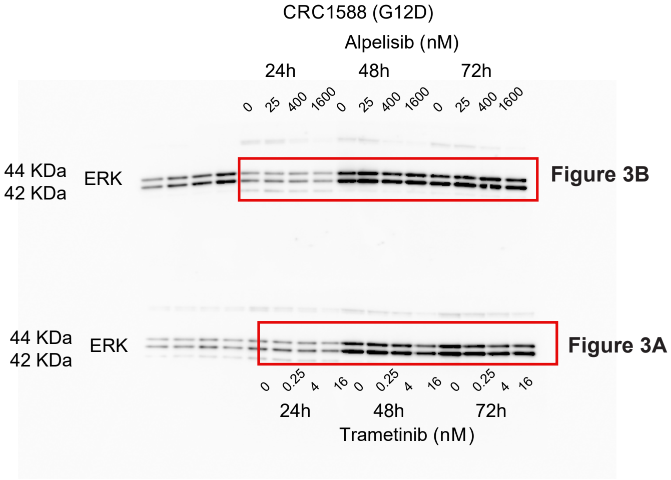
CRC1588 (G12D)
Alpelisib (nM)
24h 48h 72h
0 25 400 1600 0 25 400 1600 0 25 400 1600
44 KDa
42 KDa
ERK
Figure 3B
44 KDa
42 KDa
ERK
Figure 3A
0 0.25 4 16 0 0.25 4 16 0 0.25 4 16
24h 48h 72h
Trametinib (nM)

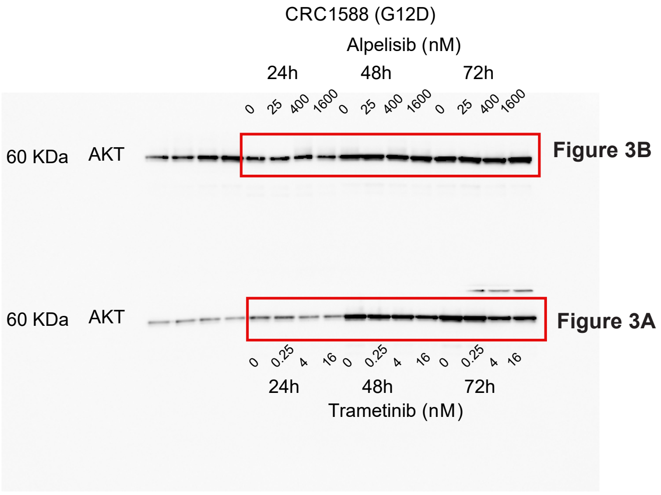
CRC1588 (G12D)
Alpelisib (nM)
24h 48h 72h
0 25 400 1600 0 25 400 1600 0 25 400 1600
60 KDa AKT
Figure 3B
60 KDa AKT
Figure 3A
0 0.25 4 16 0 0.25 4 16 0 0.25 4 16
24h 48h 72h
Trametinib (nM)

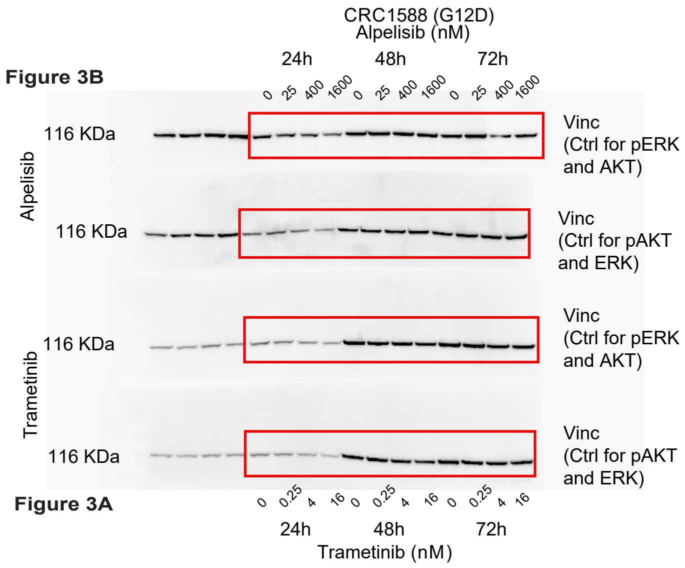
CRC1588 (G12D)
Alpelisib (nM)
24h 48h 72h
0 25 400 1600 0 25 400 1600 0 25 400 1600
Figure 3B
Alpelisib
116 KDa
Vinc (Ctrl for pERK and AKT)
116 KDa
Vinc (Ctrl for pAKT and ERK)
Trametinib
116 KDa
Vinc (Ctrl for pERK and AKT)
116 KDa
Vinc (Ctrl for pAKT and ERK)
Figure 3A
0 0.25 4 16 0 0.25 4 16 0 0.25 4 16
24h 48h 72h
Trametinib (nM)

Figure 5A

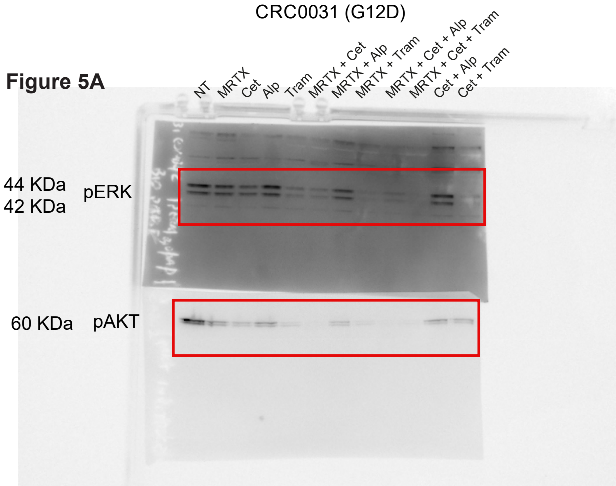

Figure 5A

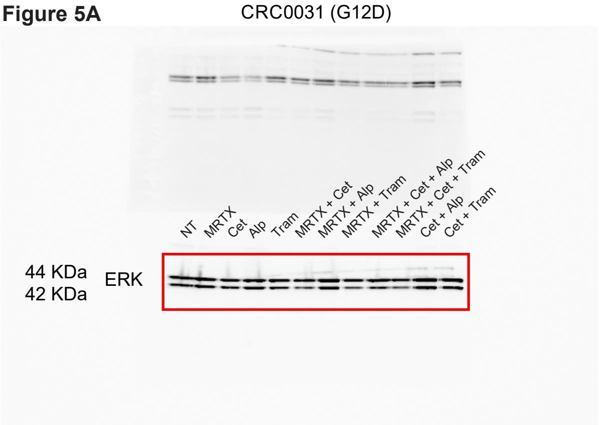

Figure 5A

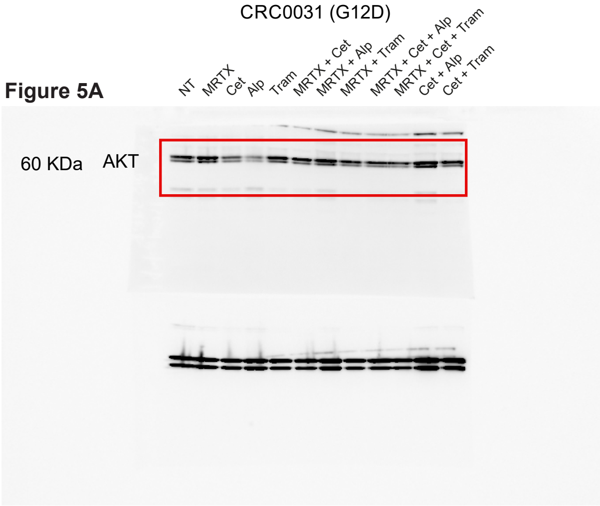

Figure 5A

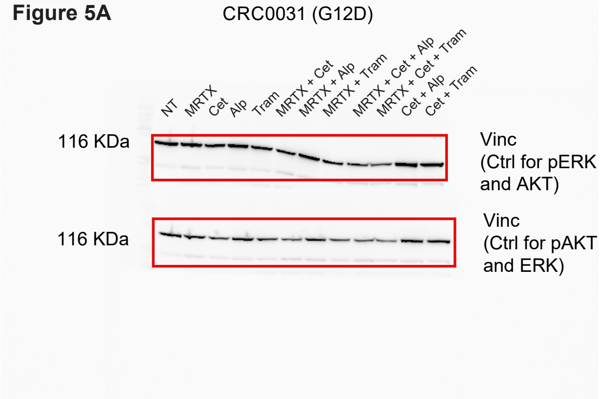

Figure 5A

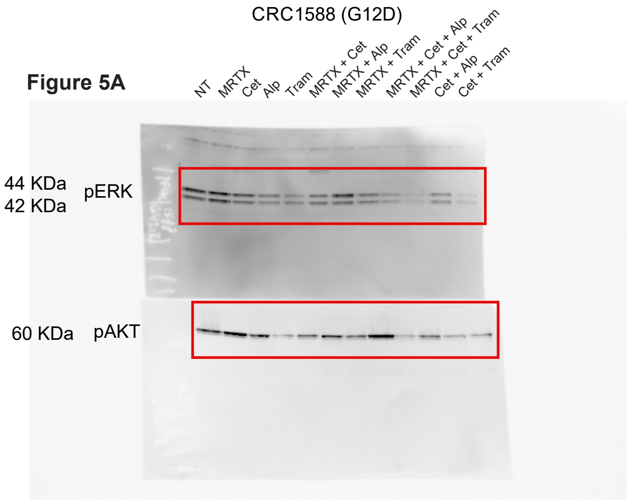

Figure 5A

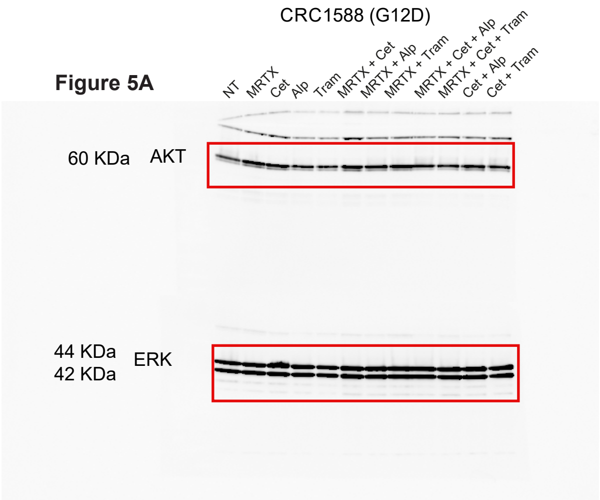

Figure 5A

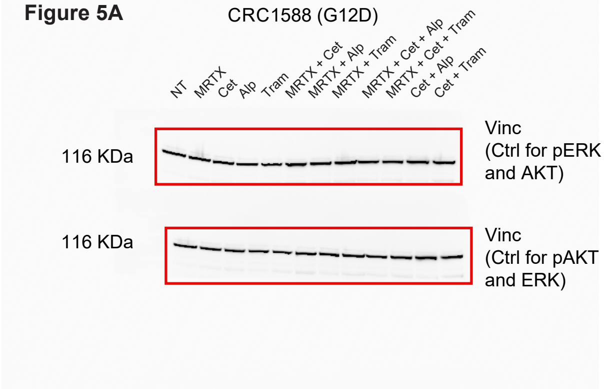

Figure 5A

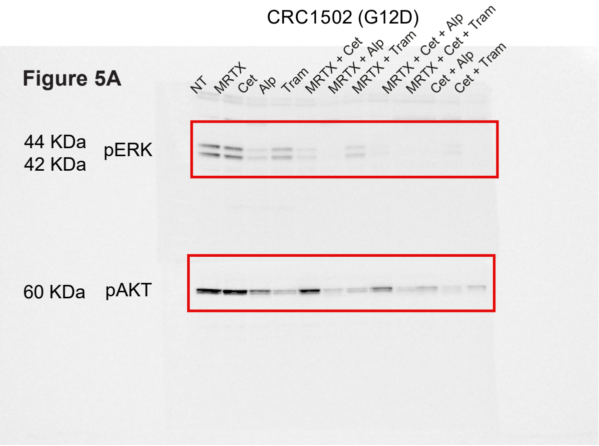

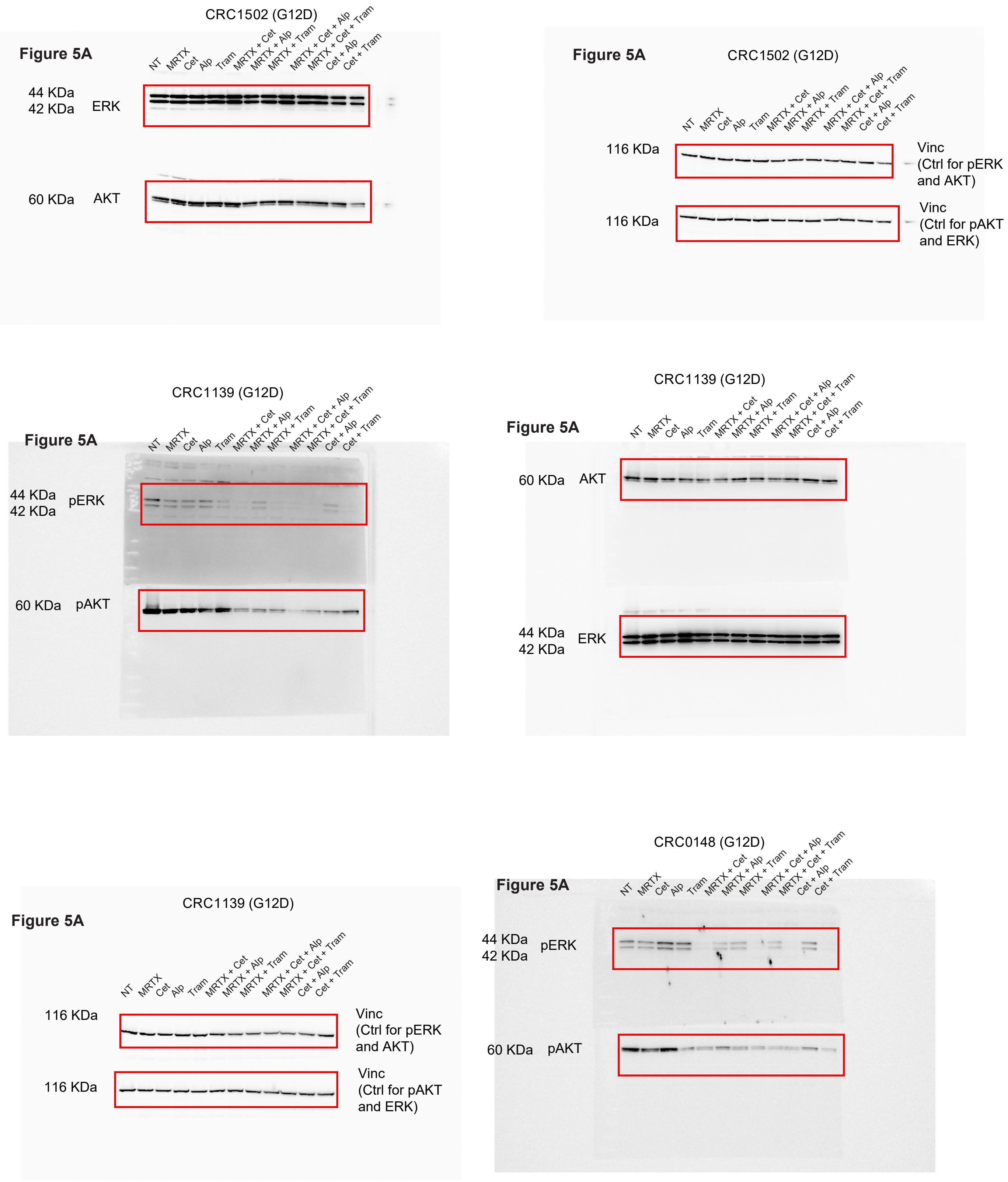

Figure 5A
CRC1502 (G12D)
NT
MRTX
Cet
Alp
Tram
MRTX + Cet
MRTX + Alp
MRTX + Tram
MRTX + Cet + Alp
MRTX + Cet + Tram
Cet + Alp
Cet + Tram
44 KDa
42 KDa
ERK
60 KDa
AKT
Figure 5A
CRC1502 (G12D)
NT
MRTX
Cet
Alp
Tram
MRTX + Cet
MRTX + Alp
MRTX + Tram
MRTX + Cet + Alp
MRTX + Cet + Tram
Cet + Alp
Cet + Tram
116 KDa
Vinc
(Ctrl for pERK and AKT)
116 KDa
Vinc
(Ctrl for pAKT and ERK)
Figure 5A
CRC1139 (G12D)
NT
MRTX
Cet
Alp
Tram
MRTX + Cet
MRTX + Alp
MRTX + Tram
MRTX + Cet + Alp
MRTX + Cet + Tram
Cet + Alp
Cet + Tram
44 KDa
42 KDa
pERK
60 KDa
pAKT
Figure 5A
CRC1139 (G12D)
NT
MRTX
Cet
Alp
Tram
MRTX + Cet
MRTX + Alp
MRTX + Tram
MRTX + Cet + Alp
MRTX + Cet + Tram
Cet + Alp
Cet + Tram
60 KDa
AKT
44 KDa
42 KDa
ERK
Figure 5A
CRC1139 (G12D)
NT
MRTX
Cet
Alp
Tram
MRTX + Cet
MRTX + Alp
MRTX + Tram
MRTX + Cet + Alp
MRTX + Cet + Tram
Cet + Alp
Cet + Tram
116 KDa
Vinc
(Ctrl for pERK and AKT)
116 KDa
Vinc
(Ctrl for pAKT and ERK)
Figure 5A
CRC0148 (G12D)
NT
MRTX
Cet
Alp
Tram
MRTX + Cet
MRTX + Alp
MRTX + Tram
MRTX + Cet + Alp
MRTX + Cet + Tram
Cet + Alp
Cet + Tram
44 KDa
42 KDa
pERK
60 KDa
pAKT

Figure 5A

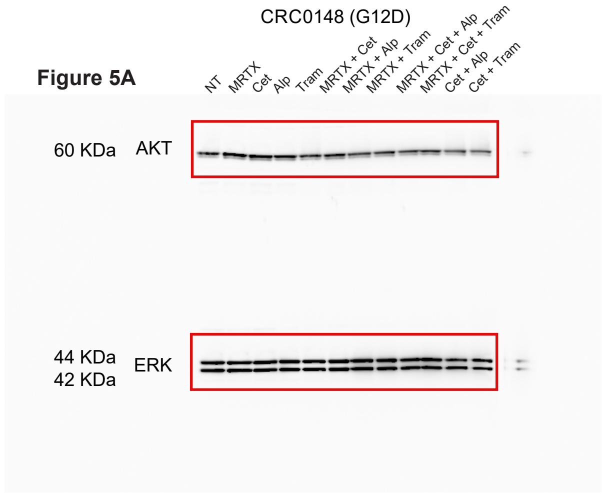

Figure 5A

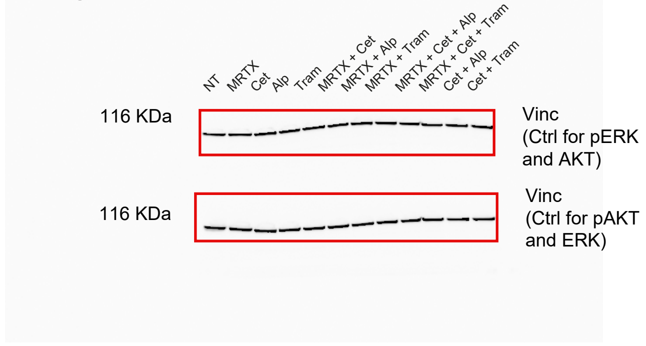

Figure 5B

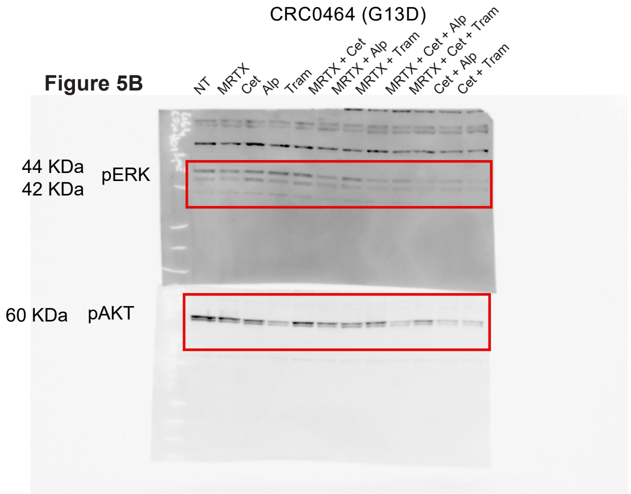

Figure 5B

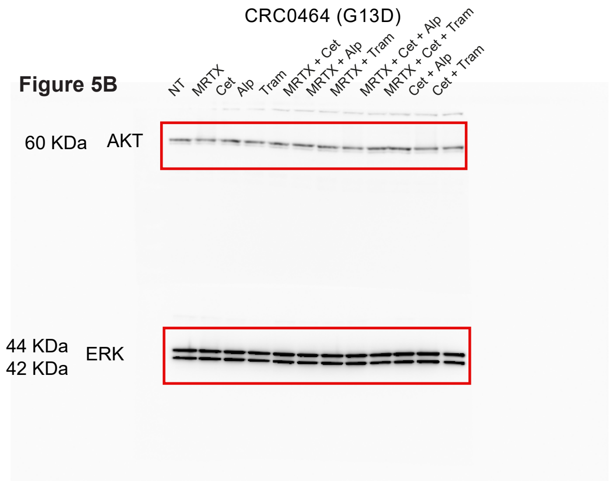

Figure 5B

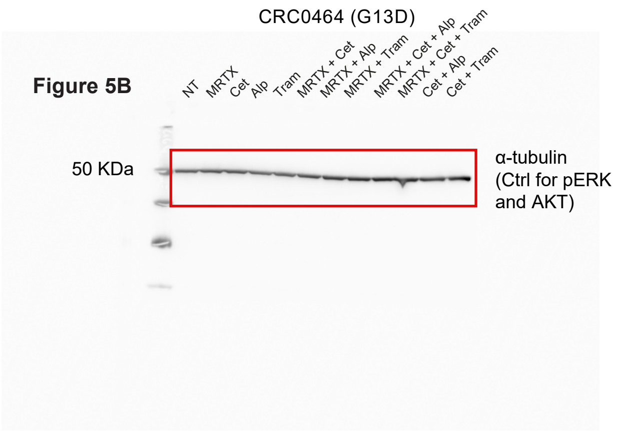

Figure 5B

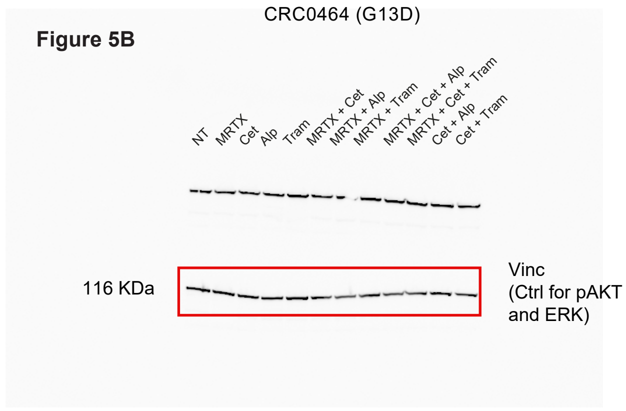

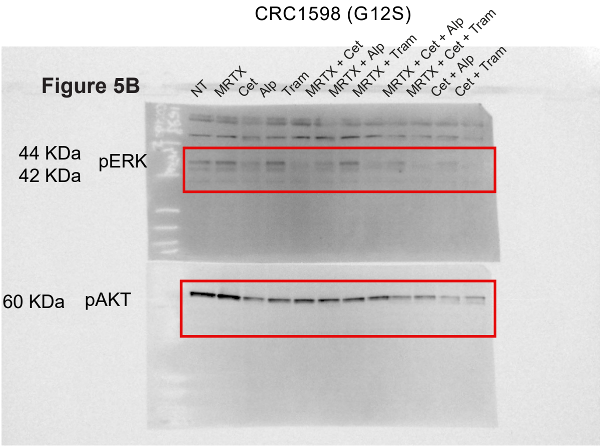
Figure 5B
CRC1598 (G12S)
NT
MRTX
Cet
Alp
Tram
MRTX + Cet
MRTX + Alp
MRTX + Tram
MRTX + Cet + Alp
MRTX + Cet + Tram
Cet + Alp
Cet + Tram
44 KDa
42 KDa
pERK
60 KDa
pAKT

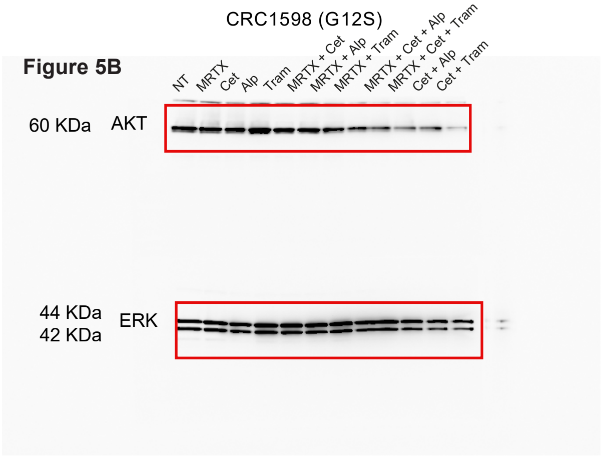
Figure 5B
CRC1598 (G12S)
NT
MRTX
Cet
Alp
Tram
MRTX + Cet
MRTX + Alp
MRTX + Tram
MRTX + Cet + Alp
MRTX + Cet + Tram
Cet + Alp
Cet + Tram
60 KDa
AKT
44 KDa
42 KDa
ERK

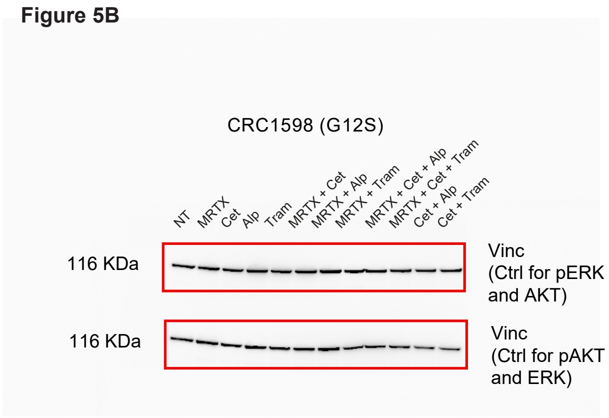
Figure 5B
CRC1598 (G12S)
NT
MRTX
Cet
Alp
Tram
MRTX + Cet
MRTX + Alp
MRTX + Tram
MRTX + Cet + Alp
MRTX + Cet + Tram
Cet + Alp
Cet + Tram
116 KDa
Vinc
(Ctrl for pERK and AKT)
116 KDa
Vinc
(Ctrl for pAKT and ERK)
